# A Study on the Corrosion Resistance and Service Life Prediction of Water-Based Epoxy-Coated Reinforced Concrete in Harsh Environments

**DOI:** 10.3390/ma19183877

**Published:** 2026-09-11

**Authors:** Zhongshuai Hu, Shaoyuan Zheng, Ping Lyu, Chunhui Zhang, Yuting Lv, Yongkang Wang, Yan Li, Xinrong Zhao, Weiqiang Zhang, Liguo Ma

**Affiliations:** 1 School of Energy and Constructional Engineering, Shandong Huayu University of Technology, Dezhou 253034, China19560659198@163.com (Y.W.); empress1232025@163.com (Y.L.); 18769617959@163.com (X.Z.); 2 Department of Civil, Construction and Environmental Engineering, Iowa State University, Ames, IA 50011, USA; 3 School of Civil Engineering, Yantai University, Yantai 264005, China

**Keywords:** water-based epoxy-coated reinforcing bars, severe environmental conditions, corrosion current density, service life, durability design of concrete structures

## Abstract

To investigate the corrosion resistance and service life of water-based epoxy-coated reinforcing bars under severe environmental conditions, HRB400 ribbed reinforcing bars were used as the substrate. Four types of water-based epoxy-coated reinforcing bars were prepared, containing 0.3% graphene–polyaniline (PAG), 0.3% iron oxide, 10% zinc phosphate, and 10% zinc–iron powder, respectively, with a bare reinforcing bar control group also included. In accordance with standards such as the ‘Design Standard for Durability of Concrete Structures’, durability tests were conducted under various conditions, including long-term immersion in marine chloride solutions, wet–dry cycling, de-icing salt freeze–thaw cycles, baking and immersion in saline soil, and concrete mixed with seawater. Corrosion current density (I_corr_) was monitored using a three-electrode system and the linear polarisation method, and service life was predicted based on the Wiener process. The results indicate that, under all severe environmental conditions, the corrosion current density of the coated reinforcing bars was significantly lower than that of the bare reinforcing bars (BRBs). After 70 cycles of marine wet–dry cycling, the corrosion current density of the bare reinforcing bars reached 0.4569 μA·cm^−2^, whilst that of the 0.3% PAG coating was 0.1103 μA·cm^−2^, substantially lower than that of the bare bars (0.4569 μA·cm^−2^); after 110 freeze–thaw cycles in a de-icing salt environment, the corrosion current density of the bare reinforcing bars was 0.4480 μA·cm^−2^, whilst that of the PAG-coated bars was 0.1003 μA·cm^−2^. After 80 cycles of baking and immersion in a saline soil environment, the corrosion current density of the graphene–polyaniline-coated steel increased from 4.97 × 10^−3^ μA·cm^−2^ to 0.1021 μA·cm^−2^ (approximately a 20-fold increase), whilst that of the bare steel rose to 0.4489 μA·cm^−2^. In concrete mixed with seawater, the corrosion current density of bare reinforcing bars reached as high as 8.60 μA·cm^−2^ after 120 days, whereas that of coated reinforcing bars was 0.24 μA·cm^−2^, markedly lower than 8.60 μA·cm^−2^ for the bare bars. Lifespan predictions indicate that, provided that the specifications for concrete strength and protective layer thickness are met, water-based epoxy coatings have the potential to delay the onset of severe corrosion (I_corr_ ≥ 1 μA·cm^−2^) beyond the 50-year design threshold in seawater wet–dry cycling zones and saline soil environments, and are projected to meet the 100-year design requirements in de-icing salt environments. It should be noted that these projections are based on accelerated tests and require validation through long-term field performance data. Graphene-containing polyaniline nanocomposite coatings exhibited the best overall protective performance, whilst zinc phosphate coatings demonstrated outstanding stability in high-chloride environments. For the specific formulations tested in this study, the enhanced corrosion resistance is attributed to the synergistic combination of the epoxy matrix, inorganic fillers (TiO_2_ and BaSO_4_) and functional additives; these components collectively provide physical shielding, chemical passivation and dynamic pore-blocking effects. Within the scope of this study, the nanocomposite coating containing 0.3 per cent PAG exhibited the best overall protective performance.

## 1. Introduction

Reinforced concrete structures have become the core structural form in modern infrastructure development due to their excellent mechanical properties and relatively low construction costs. However, in corrosive environments such as those containing marine chlorides, de-icing salts and saline soils, the corrosion of reinforcing steel within concrete has become an increasingly pressing issue and is now a key factor limiting the service life of structures [1]. When chloride ions penetrate the concrete and reach the surface of the reinforcing bars, they destroy the passivation film on the surface, inducing localised electrochemical corrosion. This, in turn, leads to a reduction in the cross-sectional area of the reinforcing bars, cracking and spalling of the concrete cover, and even a significant reduction in the structural load-bearing capacity [2]. According to statistics, the direct and indirect economic losses caused by reinforcement corrosion worldwide amount to as much as trillions of US dollars annually [3]. Consequently, how to effectively enhance the durability of reinforced concrete structures in corrosive environments has become one of the most pressing scientific challenges in the field of civil engineering materials and structures.

Among the various techniques for reinforcing bar corrosion protection, epoxy coatings are regarded as one of the most effective methods for preventing rusting of reinforcing bars and enhancing the durability of concrete structures. Traditional fusion-bonded epoxy-coated reinforcing bars (FBECR) require epoxy powder to be sprayed onto the surface of the reinforcing bars at a high temperature of approximately 232 °C; this process is energy-intensive and highly polluting, which runs counter to the development philosophy of green building materials [4]. In recent years, water-based epoxy-coated reinforcing bars have gradually become a research focus due to their environmental friendliness, ease of application, and the ability to facilitate both large-scale factory production and localised on-site application [5]. Researchers have carried out extensive work on the preparation processes of water-based epoxy coatings, filler modification, and the bond and anchorage performance of the coatings with concrete. Liu et al. systematically investigated the bond behaviour between water-based epoxy-coated reinforcing bars and concrete through 48 sets of pull-out tests, finding that the ultimate bond stress of the coated bars was approximately 10% lower than that of ordinary reinforcing bars, whilst the critical anchorage length was approximately 1.1 times that of ordinary reinforcing bars [4]. Hu et al. prepared a water-based epoxy coating system using polyamine as the curing agent and systematically investigated the effects of filler type, particle size and content on coating performance, finding that coatings containing 0.3% graphene–polyaniline composite nanomaterials exhibited excellent corrosion resistance [6]. Furthermore, Rahman et al. pointed out that epoxy coatings can simultaneously enhance both the acid corrosion resistance and bond strength of bamboo composites in concrete [7]. However, existing research has largely focused on the preparation processes and mechanical properties of the coatings themselves, with a lack of systematic evaluation of the long-term durability of water-based epoxy-coated reinforcing bars in various severe corrosive environments. At the same time, the synergistic protective mechanisms of different functional fillers (such as nanocomposites, zinc phosphate, iron(III) oxide, and zinc–iron powder) in water-based epoxy coatings are not yet fully understood. Furthermore, research into the electrochemical corrosion behaviour and service life prediction of coated reinforced concrete under complex conditions—such as chloride–sulphate coupled corrosion and freeze–thaw cycles—remains very limited.

Traditional fusion-bonded epoxy-coated reinforcing bars (FBECR) have been widely used for over 20 years as the primary corrosion protection strategy for reinforcing bars in concrete structures. FBECR is produced by heating the reinforcing bars to approximately 230 °C and spraying them with epoxy powder; this process is energy-intensive and environmentally polluting [8]. Despite its widespread adoption, FBECR still has several key limitations, raising concerns about its long-term effectiveness. Firstly, the coating is prone to defects such as missed areas, pinholes, and surface damage, which may occur during coating application, bending, transport, handling, and on-site concrete compaction [9]. These defects allow chloride ions to penetrate the coating and reach the steel substrate, triggering localised corrosion, which in turn leads to coating delamination and premature failure [10]. Secondly, FBECR significantly reduces the bond strength between the reinforcing bars and the concrete. Studies have shown that, depending on the diameter of the reinforcing bars, bond strength is reduced by 12–25 per cent, necessitating an increase in the anchorage length to compensate for this loss. Long-term temperature fluctuations may further weaken the bond performance [11]. Thirdly, field observations from marine bridge structures (including those in the Florida Keys and Ontario) indicate that premature corrosion and coating delamination have occurred within a service life of less than 10 years. Laboratory studies have further demonstrated that, when subjected to wet–dry cycling in chloride-contaminated concrete, FBECR may degrade at an unexpectedly rapid rate, with corrosion potentially initiating within 100 to 150 days of exposure [10]. In aged bridge decks, epoxy coatings may become brittle and delaminate from the steel substrate, compromising their protective function [12].

In view of these limitations, water-based epoxy coatings have emerged as a promising alternative. Unlike FBECR, water-based epoxy systems can be applied at ambient temperature, significantly reducing energy consumption and environmental impact. They also offer the potential for on-site application and repair, better damage tolerance, and superior compatibility with concrete. These advantages form the rationale for this study, which systematically investigates the corrosion resistance and service life of water-based epoxy-coated reinforcing bars under various severe environmental conditions.

In view of this, this study focuses on steel bars coated with water-based epoxy coatings and systematically conducts durability tests on them under marine chloride environments (long-term immersion and wet–dry cycling), de-icing salt environments (salt–freeze cycling) and saline soil environments (sulphate–chloride coupled baking and immersion cycling). For the tests, concrete of various strength grades ranging from C40 to C70 was prepared using Zhonglian PI.42.5R silicate cement, S95-grade mineral powder, and fly ash. The reinforcing bars used were Φ12 mm × 80 mm HRB400 ribbed bars, each coated with one of four in-house-formulated water-based epoxy coatings (containing PAG nanocomposites, nano-iron oxide, zinc phosphate and zinc–iron powder fillers), and compared with bare reinforcing bars. A Princeton 2273 electrochemical workstation was employed, using a saturated calomel electrode as the reference electrode and a stainless steel sheet as the counter electrode, to test the time-dependent changes in the corrosion current density of the reinforcing steel electrodes under various operating conditions via the linear polarisation method. On this basis, a corrosion degradation model for the steel bars was established using a univariate Wiener random process; the degradation parameters were identified using the maximum likelihood estimation method to predict the service life of coated reinforced concrete in various corrosive environments. This study aims to elucidate the protective performance and failure mechanisms of water-based epoxy coatings containing different functional fillers under severe environmental conditions, and to evaluate the suitability of water-based epoxy-coated reinforcing bars in seawater-blended concrete, with a view to providing scientific evidence and data support for the engineering application of water-based epoxy-coated reinforcing bars in marine engineering, de-icing salt environments and concrete structures in saline soil areas.

It should be noted that the five coating formulations tested in this study (Groups 1–4 in Section 2, as well as bare reinforcing bars as Group 5) were adopted from our previously published research [6], which primarily focused on the preparation process, mechanical properties and short-term corrosion resistance of these coatings in a 3.5 per cent NaCl solution. This paper represents a significant extension of that work in the following aspects. Firstly, we systematically investigated the long-term durability of the same coating formulations under four simulated severe environments that more closely resemble actual engineering service conditions: marine chloride environments (long-term immersion and wet–dry cycling), de-icing salt environments (salt–freeze cycling), saline soil environments (sulphate–chloride coupled immersion–drying cycling) and seawater-cured concrete. Secondly, we employed a three-electrode system in conjunction with linear polarisation to monitor the time-dependent behaviour of the corrosion current density (I_corr_) under various conditions, thereby providing quantitative data on the protective performance of the coating under long-term exposure conditions. Thirdly, we established a corrosion degradation model based on a univariate Wiener random process and utilised maximum likelihood estimation to predict the service life of coated reinforced concrete in various corrosive environments. These electrochemical characterisations under complex coupled service conditions, together with the Wiener process-based service life predictions, constitute the novel contributions of this study, which have not been previously reported in Reference [6] or elsewhere. The coating formulations, preparation processes and mechanical property data reported in Reference [6] have been explicitly cited in this paper and distinguished from the new experimental results presented herein.

## 2. Experiment

### 2.1. Materials

This study utilised Zhonglian P·I 42.5R Portland cement as the cementitious material (sourced from Jiuqi Building Materials Co., Ltd., Zhucheng City, Weifang City, Shandong Province, China), which has a specific surface area of 356 m^2^/kg and a density of 3.10 g/cm^3^. Its main oxide composition is as follows: CaO 62.35 per cent, SiO_2_ 21.08 per cent, Al_2_O_3_ 5.62 per cent, Fe_2_O_3_ 3.41 per cent, MgO 2.15 per cent, and SO_3_ 2.38 per cent. The S95-grade mineral powder (sourced from the Qiangdong Mineral Products Processing Plant in Lingshou County, Shijiazhuang City, Hebei Province, China) had a specific surface area of 423 m^2^/kg, a density of 2.88 g/cm^3^, a CaO content of 35.42 per cent, and a SiO_2_ content of 31.56 per cent. The fly ash is Grade II low-calcium fly ash (sourced from Yulin Refractory Materials Co., Ltd., Gongyi City, Zhengzhou, Henan Province, China), with a specific surface area of 385 m^2^/kg, a density of 2.45 g/cm^3^, a loss on ignition of 4.21 per cent, and a fineness (residue on the 45 μm sieve) of 18.5 per cent. Natural river sand was used as the fine aggregate (sourced from Dawei Building Materials Shop, Huicheng District, Huizhou City, Guangdong Province, China), with a fineness modulus of 3.65 and an apparent density of 2.62 g/cm^3^; 5–25 mm continuously graded crushed stone was used as the coarse aggregate, with an apparent density of 2.71 g/cm^3^. All cementitious materials and aggregates complied with the requirements of the relevant Chinese standards. The reinforcing bars used in the tests were HRB400 ribbed bars (sourced from Canhao Building Materials Co., Ltd., in Dezhou, China), 12 mm in diameter and 80 mm in length; their main chemical composition is shown in Table 1.

The water-based epoxy coating formulations used in this study were prepared in accordance with the procedures detailed in our previous work [6]. In brief, a water-based epoxy curing agent was prepared using polyamines (mixed amines) as the raw material (sourced from Henan Hongcheng New Materials Technology Co., Ltd., in Puyang, China). This curing agent was blended with a polyurethane-toughened water-based epoxy emulsion in a specific ratio to formulate the epoxy coating. The water-based epoxy emulsion and the curing agent were mixed in a specific mass ratio in a constant-temperature water bath at 25 ± 1 °C and stirred until homogeneous. Functional additives, such as levelling agents and defoamers (sourced from Jiezhiyuan Water Treatment Materials Co., Ltd., Gongyi City, Zhengzhou, China), were added simultaneously to eliminate microbubbles generated during mixing and to improve the rheological properties of the system. The curing agent ratio was adjusted and the curing process was optimised by testing the tensile properties of the coating film. Under the optimised curing conditions, the coating achieved a tensile strength of 40.11 MPa and an elongation at break of 19.94 per cent. Based on the results of preliminary screening, a multi-layer composite coating system was designed: a water-based epoxy coating containing a silane solution was used as the primer; a water-based epoxy coating containing 1% by mass of silane coupling agent and 10% by mass of zinc phosphate was used as the intermediate coat; and a water-based epoxy coating containing fly ash microspheres and polystyrene microspheres was used as the topcoat. The corrosion resistance of the zinc phosphate composite coating (corrosion current density of 0.00589 μA/cm^2^ after 60 days’ immersion in a 3.5% NaCl solution) was comparable to that of the 0.3% graphene/polyaniline coating. The fly ash microsphere topcoat significantly improved the deformation coordination between the reinforcing bars and the coating; the coordinated initial elongation of the multi-layer coated reinforcing bars reached 22.62 per cent, whilst the longitudinal strain at the crack reached 10.33 per cent.

Prior to electrochemical testing, coating thickness measurements and coating continuity tests were carried out on the coated reinforcing bars to ensure coating quality. For detailed preparation procedures, including curing agent synthesis, filler incorporation and multi-layer coating application, see Reference [6] and its supplementary material. It should be noted that certain process parameters—such as filler particle size and source, precise dry film thickness values, and adhesion strength—were not reported in Reference [6] and therefore cannot be provided in this study; these parameters will be systematically characterised in subsequent research.

The criteria for selecting the four types of functional fillers in this study are as follows. Firstly, in previous work, a base formulation was established using a self-prepared water-based epoxy emulsion as the matrix, with titanium dioxide (5 per cent by mass of the emulsion) and precipitated barium sulphate (10 per cent by mass of the emulsion) as essential fillers [6]. Titanium dioxide significantly enhances the coating’s corrosion resistance whilst having minimal impact on its mechanical properties, while precipitated barium sulphate is used to improve the coating’s density. Furthermore, the formulation includes additives such as wetting agents, dispersants, defoamers, and phthalocyanine blue pigment; the specific preparation process is detailed in Reference [6]. Building upon the aforementioned baseline formulation, in order to systematically compare the synergistic effects of fillers with different anti-corrosion mechanisms and to explore low-cost alternatives to expensive polyaniline/graphene nanocomposites, this study selected four representative functional fillers: (1) 0.3% graphene–polyaniline (PAG) nanocomposite, serving as a high-performance reference control—the two-dimensional sheet structure of graphene provides a physical maze barrier, whilst the emerald salt form of polyaniline forms a chemical passivation layer on the steel reinforcement surface via redox reactions; (2) 0.3% nano-Fe_3_O_4_, used to evaluate the physical sealing effect of rigid nanoparticles on coating micro-pores and their role in enhancing interface integrity; (3) 10% zinc phosphate, an environmentally friendly active pigment which, by releasing Zn^2+^ and PO_4_^3−^ ions to react with corrosion products and form insoluble passivation films such as FePO_4_, achieves dual active and passive protection; (4) 10% zinc–iron powder, used to verify the cathodic protection effect of a sacrificial anode. The aforementioned four fillers respectively encompass four typical anti-corrosion mechanisms: physical shielding, physical blocking, chemical passivation and sacrificial anodes. At the same time, they form a complete sequence across a cost gradient, ranging from high-performance PAG to economical zinc phosphate and zinc–iron powder, thereby providing an experimental basis for the selection of optimal materials to meet different engineering requirements.

The coated reinforcing bars in this experiment were divided into five groups (as shown in Table 2), namely: EP-Ti-Ba-0.3%PAG-coated reinforcing bars (Group 1), EP-Ti-Ba-0.3%Fe_3_O_4_-coated reinforcing bars (Group 2), EP-Ti-Ba-10%Zn3(PO4)2-1%KH550-coated reinforcing bars (Group 3), EP-Ti-Ba-10%Zn/Fe-1%KH550-coated reinforcing bars (Group 4), and bare reinforcing bars (Group 5). The artificial seawater solution was prepared in accordance with Table 3 [13].

It should be noted that the formulation design in this study focuses on combinations of modifiers with practical application potential, rather than a comparison of single-variable systems in the strict sense. Consequently, the comparison between Groups 1 and 2 (0.3 per cent filler, without KH550) and Groups 3 and 4 (10 per cent filler, containing 1 per cent KH550) involves simultaneous variations in filler type, filler loading, and the presence or absence of a silane coupling agent. Therefore, the observed differences in performance represent the combined effects of the entire formulation system, rather than the intrinsic properties of the filler itself. The discussion and conclusions in this paper are strictly limited to the five specific coating formulations tested in this study and do not attempt to attribute the results solely to the chemical properties of the fillers without taking these confounding factors into account. Furthermore, the mechanistic explanations and protective mechanisms discussed in the following sections (such as the physical barrier effect, electrochemical passivation, pore blockage by corrosion products, and sacrificial anode protection) are presented as hypotheses supported by the literature, with the aim of explaining the performance differences observed between the tested formulations. This study did not conduct direct experimental validation of these mechanisms—for example, via scanning electron microscopy–energy dispersive spectroscopy (SEM–EDS), X-ray diffraction (XRD), Raman spectroscopy, X-ray photoelectron spectroscopy (XPS) or coating cross-sectional analysis. Consequently, these explanations should be regarded as preliminary interpretations rather than definitive conclusions, and require further investigation in future research.

### 2.2. Preparation of Test Specimens

In accordance with standard GB/T 25826-2022 [14], the reinforcing steel working electrodes used in the tests were fabricated from HRB400 ribbed reinforcing bars, measuring Φ12 mm × 80 mm. Epoxy adhesive was applied to both ends of the epoxy-coated reinforcing bars to ensure that the working surface length of each bar was 50 mm.

#### Preparation of Concrete Test Specimens

The concrete test specimens measured 100 mm × 100 mm × 100 mm. Two HRB400 ribbed steel bars (Φ12 mm × 80 mm) were embedded parallel to one another in each specimen, with a centre-to-centre distance of 50 mm (clear distance from the outer surface: 38 mm), to ensure that both steel bars were subjected to similar diffusion conditions when the corrosive medium penetrated unidirectionally from the exposed surface. A stainless steel counter electrode (12 mm × 50 mm) was positioned parallel to the reinforcing bars, centred between them and approximately 60 mm from the top surface of the specimen. A 15 mm section at each end of each reinforcing bar was sealed with epoxy resin, while the central 50 mm section served as the exposed working electrode to ensure a constant corrosion current test area. The protective layer thickness c is defined as the shortest distance from the outer surface of the reinforcing bar to the nearest concrete surface (i.e., the thickness of the concrete layer between the top surface of the reinforcing bar and the exposed surface of the test specimen); the values for each group are 40, 45, 50, 55, 60 and 65 mm (see Section 3 for details). For the wet–dry cycling and salt–freeze tests, the top surface of the test specimen served as the unidirectional exposure surface to the corrosive medium, whilst the remaining five surfaces were sealed with epoxy resin or aluminium foil tape to ensure that the corrosive medium penetrated only from the top surface, thereby simulating the unidirectional corrosion conditions experienced by structures in actual engineering applications. Electrical connections were established by soldering copper wires to the sealed ends of the reinforcing bars and routing them through the side formwork; all exposed wire joints were wrapped with insulating tape to prevent the formation of an electrical circuit with the external medium. After moulding, the test specimens were demoulded and subjected to standard curing in accordance with GB/T 50081-2019 [15], with a curing period of 28 days. This configuration was maintained consistently across all subsequent test series; only the protective layer thickness and exposure conditions were varied in accordance with the test design.

The concrete strength class and water-to-binder ratio were determined according to the different corrosion environments. For each environmental exposure condition, five independent concrete test blocks (cubes) were prepared for each of the five reinforcement groups (four coated types and one bare control), i.e., a sample size of n = 5 independent specimens per group. Two reinforcing bars were embedded in each cube to provide technical replicates for the electrochemical measurements. The average of the readings from the two bars within the same cube was used as the representative value for that independent specimen to avoid pseudoreplication. All data presented in the results are expressed as mean ± standard deviation derived from the five independent specimens. The concrete mix design is shown in Table 4. The required materials were weighed out in accordance with Table 4 to form the concrete specimens, into which stainless steel plates measuring 12 mm × 50 mm were inserted to serve as counter electrodes. After moulding, the specimens were cured for 28 days. It should be noted that this study did not measure the fresh concrete properties (slump), air content or hardened density, as its primary objective was to evaluate electrochemical corrosion behaviour and long-term durability, rather than the workability or physical properties of the concrete. These parameters will be systematically characterised in subsequent work to provide a more comprehensive assessment of the concrete’s performance.

## 3. Test Methods

To investigate the durability of coated reinforced concrete under different operating conditions and environments, this experiment was based on the classification of concrete service environment exposure grades set out in standard GB/T 50476-2019 [16] and standard T/CECS1203-2022 [17]. Four simulated environments and a variety of concrete design conditions were devised to investigate the durability of water-based epoxy-coated reinforced concrete under severe environmental conditions.

### 3.1. Test Methods for Assessing the Durability of Coated Reinforced Concrete in Marine Chloride Environments

GB/T 50476-2019 sets out the environmental exposure classes for marine chloride environments, as shown in Table 5. Based on the descriptions of environmental conditions in the table, wet–dry cycle tests were conducted in accordance with Class III-F to assess the durability performance of coated reinforced concrete under periodic tidal or wave splash conditions.

GB/T 50476-2019 specifies the minimum thickness of the protective layer for concrete materials and reinforcing steel in chloride environments, as shown in Table 5 below. In this test, concrete specimens were designed for the two design lifetimes (50 years and 100 years) of Class III-F environments in accordance with Table 6. The test programme for the durability of coated reinforced concrete in marine chloride environments is shown in Table 7.

In order to simulate the wet–dry cycling process of reinforced concrete in a natural environment, the experiment divided the year into three periods, with simulations carried out in each period according to the temperatures and wet-to-dry ratios specified in Table 8. After the test specimens had been immersed in a water bath at room temperature for a certain period, they were placed in a TH-4P-B programmable constant temperature and humidity test chamber for drying. The test consisted of one cycle every three days, with electrochemical testing carried out after every five cycles.

### 3.2. Test Protocol for the Durability of Coated Reinforced Concrete in De-Icing Salt Environments

With reference to Table 6.2.5 in the standard GB/T 50476-2019 [16] and Table 5.2.2 in T/CECS 1202-2022 [18], the environmental exposure classes for de-icing salts are categorised as shown in Table 9.

The test programme for the durability of epoxy-coated reinforced concrete in de-icing salt environments was designed in accordance with Table 6 and Table 9, as shown in Table 10. The curing regime for the test specimens was determined in accordance with JTG 3420-2020 [19]. Five reinforced concrete test specimens were cast for each group using solutions of different chloride ion concentrations. After moulding, the specimens were cured in their moulds for 24 h ± 2 h, then transferred to a standard curing chamber for curing until day 22, at which point they were removed and dried. Following drying, the specimens were sealed; on day 24, the test surfaces were saturated with water, and on day 28, the specimens underwent salt–freeze testing.

The freeze–thaw cycle regime was designed in accordance with the provisions of T/0583-2020 [20] ‘Test Method for Salt-Induced Freeze Resistance of Cement Concrete (Single-Face Method)’ in JTG 3420-2020 [19]. The test specimens were subjected to a cycle consisting of 12 h at –20 °C followed by 12 h at 20 °C; this constituted one cycle, and electrochemical testing was carried out after every five cycles.

### 3.3. Test Protocol for the Durability of Coated Reinforced Concrete in Saline Soil Environments

In environments characterised by high concentrations of coupled sulphate and chloride corrosion, both types of corrosive agents migrate into the concrete; sulphates cause corrosion and spalling of the concrete cover, while the migration of chlorides and the spalling of the protective layer accelerate the corrosion of the reinforcing steel. This coupled degradation process significantly accelerates the deterioration of concrete performance. In actual service environments, sulphates and chlorides typically coexist; therefore, it is of great importance to investigate the durability of reinforced concrete structures or components coated with water-based epoxy coatings in chloride–sulphate coupled environments. Reference standard T/CECS 1203-2022 [17] and the Technical Specification for Application T/CECS 1202-2022 [18] classify the severity levels of saline soil environments as shown in Table 11.

The Design Standard for the Durability of Concrete Structures in Harsh Environments (T/CECS 1203-2022) [17] specifies the minimum concrete strength class, maximum water-to-cement ratio, and minimum cover thickness for concrete in saline soil environments subject to high-concentration sulphate–chloride coupled corrosion, as shown in Table 12.

Based on Table 11 and Table 12, a drying-immersion test programme for saline soil environments was formulated, as shown in Table 13. The test protocol for the drying-immersion cycles is as follows: reinforced concrete specimens are dried in an oven at 60 °C for 24 h; to avoid thermal stress caused by sudden temperature changes, the specimens must be allowed to cool at room temperature for 3 h before being immersed in the corrosive solution for 45 h. Each 3-day period constitutes a complete drying-immersion cycle. For coated reinforced concrete specimens undergoing drying-immersion cycles, electrochemical performance tests are carried out after every 5 cycles.

The specific configuration of the electrochemical testing system used in the experiment is described below. In accordance with standard [21], a Princeton Model 2273 workstation was employed for corrosion current density testing. A conventional three-electrode configuration was adopted, with a saturated calomel electrode (SCE) as the reference electrode, a stainless-steel plate (12 mm × 50 mm) as the counter electrode, and the coated reinforcing bars embedded in concrete as the working electrode. An open-circuit potential of 10 mV was set as the parameter for the polarisation curve test, and all test specimens were required to be fully immersed in a 3.5% NaCl solution for measurement.

The linear polarisation method is considered appropriate for assessing the degree of reinforcement corrosion. This method is characterised by its remarkable ease of operation, notable speed of testing and high sensitivity. It offers significant advantages in the field of non-destructive testing. Practical examples demonstrate that this method is more practical than traditional methods in terms of the convenience of analysing results.

After the corrosion current density has been measured, the relationship between the corrosion current density (I_corr_) and the degree of corrosion should be determined in accordance with the Broomfield criteria [22] (Table 14).

### 3.4. Electrochemical Testing Method

The electrochemical measurements were performed using a Princeton Model 2273 potentiostat/galvanostat. A conventional three-electrode configuration was adopted, with a saturated calomel electrode (SCE) as the reference electrode, a stainless-steel plate (12 mm × 50 mm) as the counter electrode, and the coated reinforcing bar embedded in concrete as the working electrode.

Prior to each measurement, the open-circuit potential (OCP) was monitored until it stabilised. Stabilisation was assumed when the potential drift was less than ±1 mV/min, which typically required 30 min (1800 s). The potentiodynamic polarisation scan was then performed from −10 mV to +10 mV versus the stabilised OCP at a scan rate of 0.1667 mV/s, in accordance with ASTM G59-97(2014) Standard Test Method for Conducting Potentiodynamic Polarisation Resistance Measurements [21]. The polarisation resistance, Rp, was determined as the slope of the potential-vs-current density plot at the zero-current point. The corrosion current density I_corr_ was calculated from the Stern–Geary relationship: I_corr_ = B/Rp, where B is the Stern–Geary coefficient. Following the RILEM TC 154-EMC recommendations [23], B was taken as 26 mV for steel in the active corrosion state and 52 mV for steel in the passive state.

The exposed working area of each reinforcing bar was the cylindrical surface of the 50 mm middle section (bar diameter 12 mm; exposed area ≈ 18.85 cm^2^ per bar). All reported I_corr_ values were normalised by this area. The ohmic drop (IR) correction was not applied to Rp because the specimens were fully immersed in 3.5% NaCl solution and the current density was consistently below 1 μA/cm^2^, making the IR drop negligible (<0.1 mV). The reference electrode was placed approximately 10 mm from the working electrode surface within the same electrolyte bath. Each of the two reinforcing bars in each cube specimen was measured independently, and the I_corr_ value reported for each test group represents the average of the two bars. For each test condition, five replicate specimens were prepared and tested; the relative standard deviation (RSD) of the I_corr_ measurements was consistently below 5%, confirming acceptable repeatability. The linear polarisation method is considered appropriate for assessing reinforcement corrosion owing to its operational simplicity, high sensitivity, and suitability for nondestructive evaluation.

After measuring I_corr_, the corrosion state of the reinforcement was classified according to the Broomfield criteria [24] (Table 14), which are consistent with the threshold criteria recommended by RILEM TC 154-EMC [23] (I_corr_ < 0.1 μA·cm^−2^ for the passive state and I_corr_ > 1 μA·cm^−2^ for severe corrosion). All electrochemical tests were conducted with the specimens fully immersed in 3.5% NaCl solution. The exposure conditions for each test series are summarised in Table 7, Table 10 and Table 13.

### 3.5. Method for Determining the Service Life of Coated Reinforced Concrete

Many researchers predict the service life of reinforced concrete members based on concrete mass loss or changes in the dynamic elastic modulus; however, it is the corrosion of reinforcing steel that is the key factor affecting structural durability. Traditional prediction methods based on the chloride ion diffusion equation assume that the diffusion process is uniform; however, this assumption does not apply to real-world conditions in complex environments. Furthermore, some researchers have estimated the extent of reinforcement corrosion and the residual service life of structures based on the width of corrosion-induced cracks or the ultimate load-bearing capacity [25,26,27]. In contrast, electrochemical testing provides a non-destructive and convenient method for on-site inspection. By measuring the corrosion current density, it not only reflects the state of reinforcement corrosion but also enables the accurate calculation of the instantaneous corrosion rate.

The Wiener process {X(t), t ≥ 0} is characterised by independent and normally distributed increments with X(0) = 0. For a standard Wiener degradation process with constant drift μ, the degradation X(t) = μt + σB(t) has variance Var [X(t)] = σ^2^t, which increases linearly with time. The mean degradation rate remains constant at μ and does not automatically accelerate. However, the process has non-monotonic sample paths and, under the assumed positive drift μ > 0, the probability of eventually reaching a specified failure threshold l approaches 1 as t → ∞ [28]. In this study, X(t) is defined as the corrosion current density I_corr_ (in μA·cm^−2^) measured in the t-th cycle. The initial value X(0) is taken as 0 (corresponding to the corrosion current density of fully passivated reinforcing bars). The failure threshold l is set at 1.0 μA·cm^−2^; according to the Bloomfield standard (Table 14), this value corresponds to the initial stage of severe corrosion. Therefore, the service life T of a product is defined as the time at which the performance degradation first reaches *l*.

It should be emphasised that the failure threshold *l* = 1.0 μA·cm^−2^ corresponds to the onset of severe corrosion as defined by the Broomfield criteria (Table 14) and the RILEM TC 154-EMC recommendations. This threshold represents a warning indicator for the need for maintenance or repair actions, rather than the ultimate structural failure (e.g., loss of load-bearing capacity, corrosion-induced cracking, or spalling). A more rigorous determination of structural service life would require the integration of the time-dependent corrosion current density to estimate cumulative metal loss (e.g., using Faraday’s law) and its correlation with a physical limit state, as recommended in ASTM G225-26 [29]. However, such an analysis was beyond the scope of the present study, which focuses on comparative evaluation of coating performance. Therefore, the predictions presented herein should be interpreted as the “time to reach the high-corrosion-rate threshold” rather than the absolute structural service life.

(1) $$\begin{array}{c} T=\inf\{t\left|X\left(t\right)=l,t\geq 0\right\} \end{array}$$

From Equation (1), it can be derived that the distribution of the lifetime *T* is an inverse Gaussian distribution. The basic derivation is as follows:

Based on X(t), define the random process {*Z*(*t*), *t* ≥ 0} as shown in Equation (2).

(2) $$\begin{array}{c} Z\left(t\right)=\underset{0\leq s\leq t}{\sup}\left\{X\left(s\right),s\geq 0\right\} \end{array}$$

Let *Z*(*t*) denote the maximum value of *X*(*t*) over the time interval [0, t) for any time *t* (*t* ≥ 0). Let *g*(*z*, *t*) denote the probability density function of *Z*(*t*) at time t; then, the probability that the product has not failed by time t is given by Equation (3):

(3) $$\begin{array}{c} P\left\{T>t\right\}=P\left\{Z\left(t\right)<l\right\}=\int_{-\infty }^{l}g\left(z,t\right)dz \end{array}$$

In the Fokker–Planck equation (Kolmogorov forward equation), *g*(*z*,*t*) takes the form given in Equation (4):

(4) $$\begin{array}{c} g\left(z,t\right)=\frac{1}{\sigma \sqrt{2\pi t}}\left\{\exp\left[-\frac{{\left(z-\mu t\right)}^{2}}{2\sigma^{\prime }t}\right]-\exp\left(\frac{2\;\mu L}{\sigma^{\prime }}\right)\exp\left[-\frac{{\left(z-2l-\mu t\right)}^{2}}{2\sigma^{\prime }t}\right]\right\} \end{array}$$

Substituting Equation (4) into Equation (3) yields the reliability function (5):

(5) $$\begin{array}{c} P\{T>t\}=1-F(t)=\Phi (\frac{l-\mu t}{\sigma \sqrt{t}})-\exp(\frac{2\;\mu L}{\sigma^{2}})\Phi (\frac{-l-\mu t}{\sigma \sqrt{t}}) \end{array}$$

Consequently, the distribution function and probability density function for the service life, *T*, of the product are given by Equations (6) and (7):

(6) $$\begin{array}{c} F(t)=\Phi (\frac{\mu t-l}{\sigma \sqrt{t}})+\exp(\frac{2\;\mu L}{\sigma^{2}})\Phi (\frac{-l-\mu t}{\sigma \sqrt{t}}) \end{array}$$

(7) $$\begin{array}{c} f(t)=\frac{l}{\sqrt{2\pi \sigma^{2}t^{3}}}\exp[-\frac{(l-\mu t{)}^{2}}{2\sigma^{2}t}] \end{array}$$

In Equations (6) and (7), the degradation parameters to be estimated are *μ* and *σ*^2^, and this study employs the maximum likelihood estimation method to identify the unknown parameters. The degradation increment ∆x, based on the characteristics of a univariate Wiener random process, follows a normal distribution with mean *μ* and variance *σ*^2^. Based on the properties of the normal distribution, the likelihood function for ∆x is given by Equation (8):

(8) $$\begin{array}{c} L\left(\mu ,\sigma^{2}\right)=\prod_{j=1}^{k}\frac{1}{\sqrt{2\pi \sigma^{2}\Delta t_{j}}}\exp\left[-\frac{(\Delta x_{j}-\mu \Delta t_{j}{)}^{2}}{2\sigma^{2}\Delta t_{j}}\right] \end{array}$$

Taking the logarithm of both sides of Equation (8) yields the log-likelihood function given by Equation (9):

(9) $$\begin{array}{c} \ln L(\theta )=-\frac{k}{2}\ln(2\pi )-\frac{k}{2}\ln(\sigma^{2})-\frac{1}{2}\sum_{j=1}^{k}\ln(\Delta t_{j})-\sum_{j=1}^{k}\frac{{\left(\Delta x_{j}-\mu \Delta t_{j}\right)}^{2}}{2\sigma^{2}\Delta t_{j}} \end{array}$$

By taking the partial derivatives of Equation (9), we obtain the maximum likelihood estimates for *μ* and *σ*^2^, as shown in Equations (10) and (11):

(10) $$\begin{array}{c} \mu =\frac{\sum_{j=1}^{k}\Delta x_{j}}{\sum_{j=1}^{k}\Delta t_{j}}=\frac{x_{k}}{t_{k}} \end{array}$$

(11) $$\begin{array}{c} \sigma^{2}=\frac{1}{k}\sum_{j=1}^{k}\frac{{\left(\Delta x_{j}-\mu \Delta t_{j}\right)}^{2}}{\Delta t_{j}}=\frac{1}{k}\left(\sum_{j=1}^{k}\frac{\Delta x_{j}^{2}}{\Delta t_{j}}-\frac{x_{k}^{2}}{t_{k}}\right) \end{array}$$

It is worth noting that, in accordance with the requirements of the ASTM G225-26 standard [29], the cumulative metal loss can be determined by integrating the instantaneous corrosion current density measured using the linear polarisation method over time. Using Faraday’s law, the relationship between the corrosion current density, I_corr_ (in μA·cm^−2^), and the corrosion penetration rate can be expressed as penetration rate (μm/year) = 11.6 × I_corr_ (μA·cm^−2^). Through this conversion, I_corr_ data varying over time can be converted into cumulative cross-sectional loss, which can then be compared with physical limit states (such as the critical reduction in reinforcing bar diameter or the onset of cracking), thereby enabling a more rigorous prediction of service life.

It should be acknowledged that the prediction results based on the Wiener process in Section 4.6, Section 4.7 and Section 4.8 do not include confidence intervals or individual parameter estimates (μ and σ^2^) for each operating condition; the reported lifetimes correspond to the median (50th percentile) of the inverse Gaussian distribution. Whilst this limitation does not affect the conclusions regarding the comparison of coating performance, it does restrict the scope of statistical inference. Future work will be conducted using open-source software and will systematically report all statistical parameters and confidence intervals.

## 4. Results of Durability Tests on Reinforced Concrete with Water-Based Epoxy Coatings

### 4.1. Analysis of Corrosion Current Density Results for Coated Reinforced Concrete Under Conditions of Long-Term Immersion in Seawater

**Figure 1:**
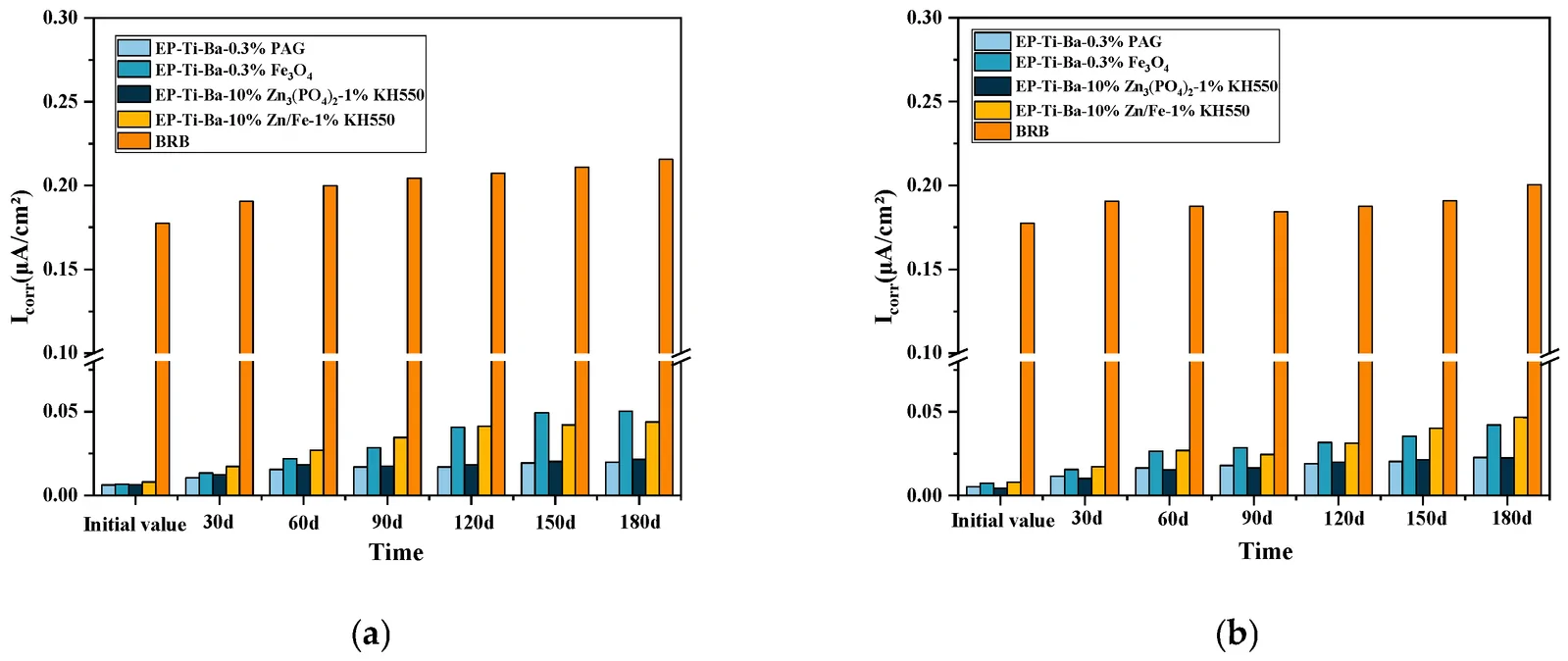
Corrosion current density results for coated reinforced concrete under conditions of prolonged immersion in seawater: (**a**) C40-40c-IIIC-50; (**b**) C45-45c-IIIC-100.

Figure 1 shows the time-dependent variation in I_corr_ for various reinforcing steel electrodes in concrete subjected to long-term immersion in artificial seawater. The figure shows that the corrosion current density of the coated steel bar electrodes is significantly lower than that of the bare steel bar electrodes. For the bare steel bar electrodes, the initial corrosion current density exceeded 0.1 μA·cm^2^ after 28 days of curing, indicating that the steel bars were no longer in a passivated state; following 180 days of prolonged immersion in artificial seawater, the corrosion current density reached approximately 0.2 μA·cm^2^. By contrast, the initial value for coated steel reinforcement was less than 0.001 μA·cm^2^, and after 180 days of prolonged immersion in artificial seawater, the corrosion current density remained below 0.05 μA·cm^2^.

### 4.2. Analysis of Corrosion Current Density Results for Coated Reinforced Concrete Under Dry–Wet Cycling Conditions

**Figure 2:**
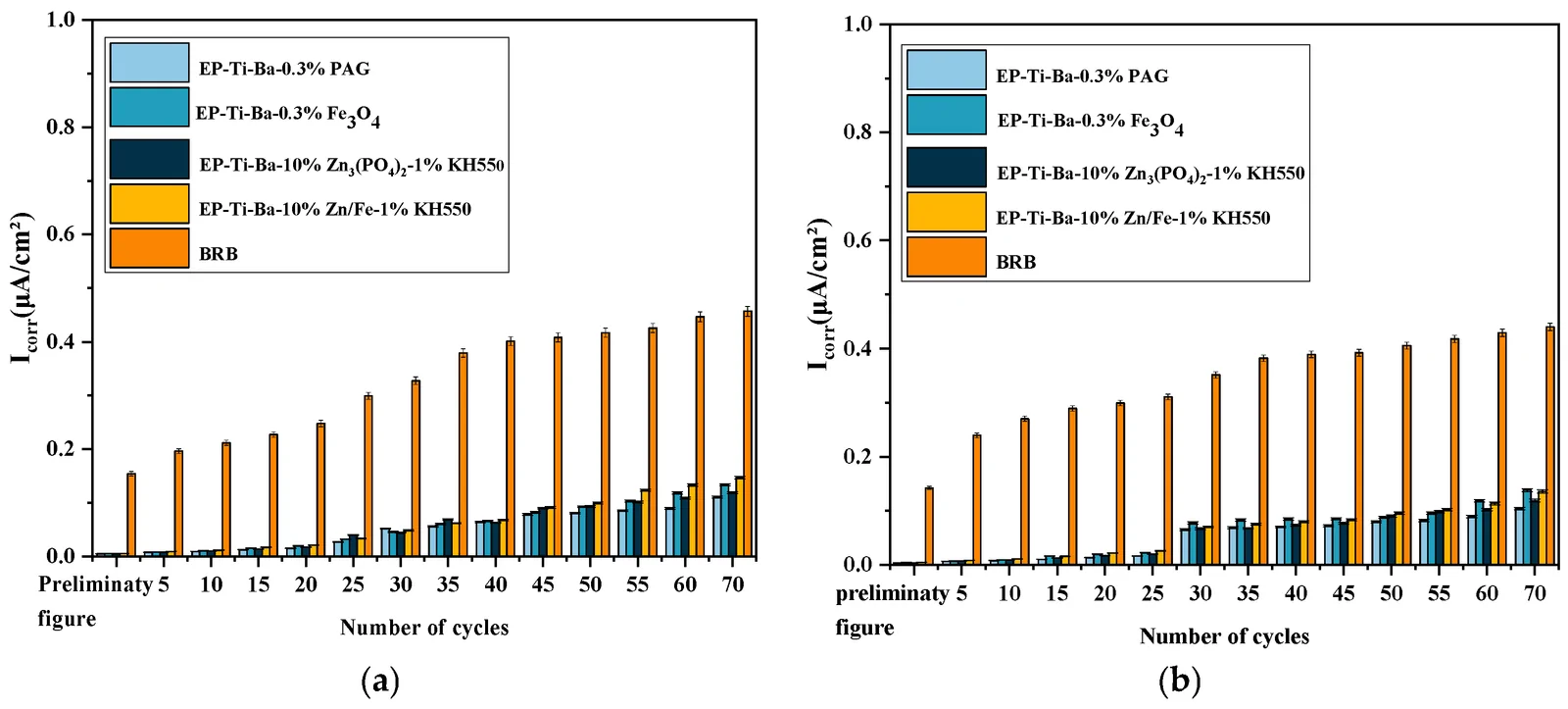
Corrosion current density results for coated reinforced concrete under wet–dry cycling conditions in seawater: (**a**) C50-60c-IIIF-50; (**b**) C50-65c-IIIF-100.

Figure 2 shows the time-dependent variation in I_corr_ for various reinforcing steel electrodes in concrete under a dry–wet cycle environment using artificial seawater. As can be seen from the figure, after 28 days of curing, the initial corrosion current density of bare reinforcing steel had already exceeded 0.1 μA·cm^2^, indicating that the steel was in a non-passivated state. The application of a coating can effectively protect the reinforcing steel, reducing the risk of corrosion and thereby improving the durability of the structure. For example, Figure 2 shows the time-dependent variation in I_corr_ for reinforcing steel electrodes under the C50-60c-IIIF-50 conditions. The initial corrosion current density of the bare reinforcing steel was 0.1541 μA·cm^2^; after 70 cycles, I_corr_ rose to 0.4569 μA·cm^2^, indicating that the reinforcing steel was in a state of low corrosion. In contrast, after 70 cycles, the I_corr_ values for all four types of coated reinforcing bars were significantly lower than that of the bare reinforcing bar, remaining below 0.15 μA·cm^2^, indicating that the coatings effectively prevented corrosion of the reinforcing bars. Among these, the corrosion current density was lowest for the coating containing 0.3% PAG nanocomposite, with an I_corr_ of 0.1103 μA·cm^−2^, whilst the corrosion current density of the coated rebar containing 10% Zn_3_(PO_4_)_2_ was the closest, with an I_corr_ of 0.1185 μA·cm^−2^.

From the perspective of the corrosion mechanism at the coating–reinforcing bar interface, the evaporation of water during the curing process of water-based epoxy coatings inevitably leads to the formation of micro-pores and conductive pathways, providing penetration routes for corrosive media such as Cl^−^. Once Cl^−^ penetrates the coating and reaches the surface of the reinforcing bar, it destroys the passivation film on the surface, triggering localised electrochemical corrosion [6]. Among the four coatings, the PAG coating significantly lengthens the diffusion path of corrosive media due to the ‘maze effect’ created by the two-dimensional layered structure of graphene; simultaneously, the emerald salt form of polyaniline can form a dense passivation layer at the coating–reinforcing bar interface via redox reactions. Consequently, its I_corr remains consistently the lowest. The zinc phosphate coating, on the other hand, forms inhibitory chemical conversion layers such as FePO_4_ and Fe_2_O_3_ on the rebar surface through the release of Zn^2+^ and PO_4_^3−^ [1,6]; its mechanism of ‘dynamic sealing of micropores by corrosion products’ ensures that I_corr remains stable during the later stages of wet–dry cycling.

### 4.3. Analysis of Corrosion Current Density Results for Coated Reinforced Concrete Under De-Icing Salt Conditions

**Figure 3:**
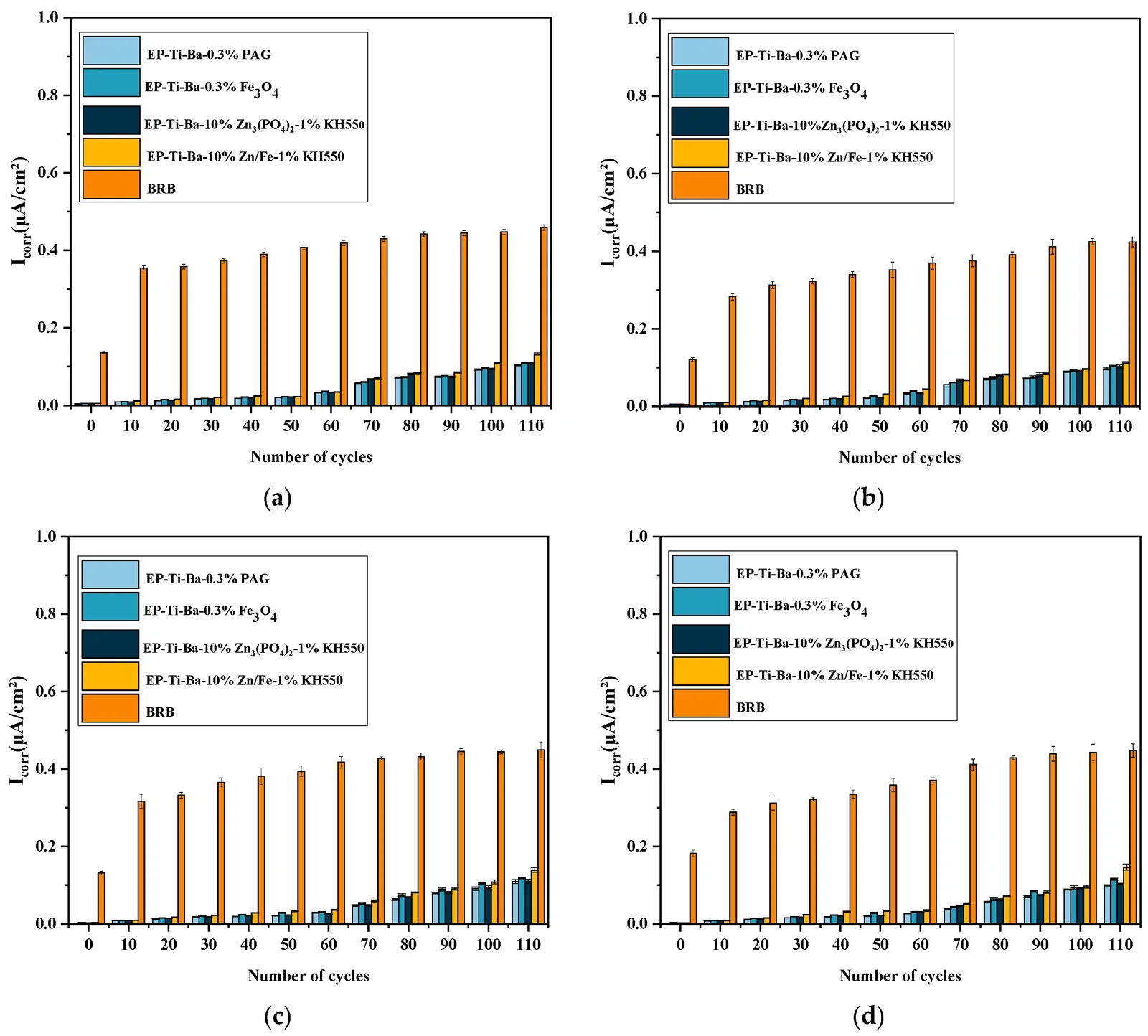

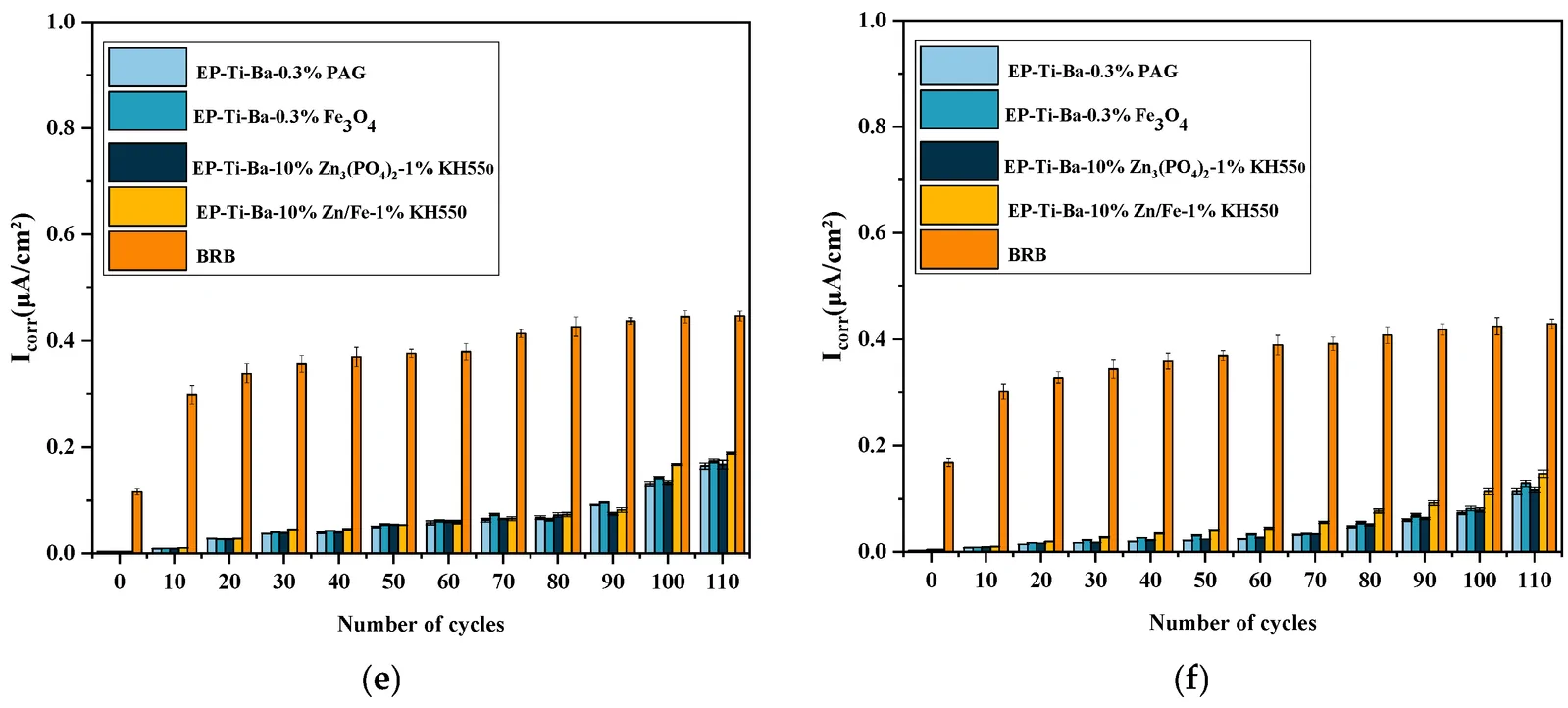
Corrosion current density results for coated reinforced concrete under de-icing salt conditions: (**a**) C45-45c-IVD-50; (**b**) C45-55c-IVD-100; (**c**) C50-50c-IVE-50; (**d**) C50-60c-IVE-100; (**e**) C50-60c-IVF-50; (**f**) C50-65c-IVF-100.

Figure 3 shows the time-dependent variation in corrosion current density of steel reinforcement electrodes in a de-icing salt environment. In the test specimen (C50-60c-IVE-100) with concrete strength C50, a cover thickness of 60 mm and a chloride ion concentration of 10,000 mg/L, after 110 freeze–thaw cycles, the corrosion current density (I_corr_) of the bare steel reinforcement electrode was 0.4480 μA/cm^2^; the PAG coating exhibited the best protective performance, with a corrosion current density (I_corr_) of 0.1003 μA/cm^2^; the excellent performance of the EP-Ti-Ba-0.3% PAG formulation may be attributed to the dense polymer structure resulting from the synergistic reinforcement provided by the PAG nanocomposite, which appears to effectively impede chloride ion penetration. However, as the PAG formulation also differs from the zinc phosphate and zinc–iron powder formulations in terms of filler content and the presence or absence of silane, this explanation remains preliminary and is limited to the formulations tested. The I_corr_ value for the zinc phosphate coating was slightly higher (0.1032 μA/cm^2^); its protective mechanism relies on corrosion products dynamically plugging micro-pores. In a chloride-rich environment, the corrosion products of zinc phosphate—zinc hydroxychloride and ferric phosphate—form a protective effect that may involve dynamic pore-blocking processes through the dual action of physical plugging and chemical passivation, thereby inhibiting ion migration. However, micro-cracks in the concrete induced by freeze–thaw cycles may still cause some deterioration in its long-term performance, which requires further experimental observation. The I_corr_ value for the zinc–iron powder coating was 0.1467 μA/cm^2^. The protective effect of the zinc–iron powder coating may involve a sacrificial anode mechanism; however, the rapid consumption of zinc in high-chloride environments appears to limit its long-term efficacy, based on the observed I_corr_ evolution.

Further verification suggested that in an environment with a higher chloride ion concentration (20,000 mg/L, test specimen C50-60c-IVF-50), the I_corr_ of the zinc phosphate coating remained stable at 0.1646 μA/cm^2^ after 110 cycles, outperforming the 0.1884 μA/cm^2^ recorded for the zinc–iron powder coating, indicating that its microporous blocking mechanism is well-suited to high-chloride environments; whereas the I_corr_ of bare reinforcing bars under the same conditions reached as high as 0.4473 μA/cm^2^, far exceeding that of all coatings, once again confirming the necessity of coating protection. In summary, the excellent stability and masking ability of PAG nanomaterials, combined with the pore-blocking protective mechanism of zinc phosphate corrosion products, ensure the durability of coated reinforcing bars in de-icing salt environments. Freeze–thaw cycles exacerbate coating failure through dual pathways: physical cracking and chemical degradation. Therefore, dynamic protective mechanisms and material stability are key to enhancing durability.

### 4.4. Analysis of Corrosion Current Density Results for Coated Reinforced Concrete in Saline Soil Environments

**Figure 4:**
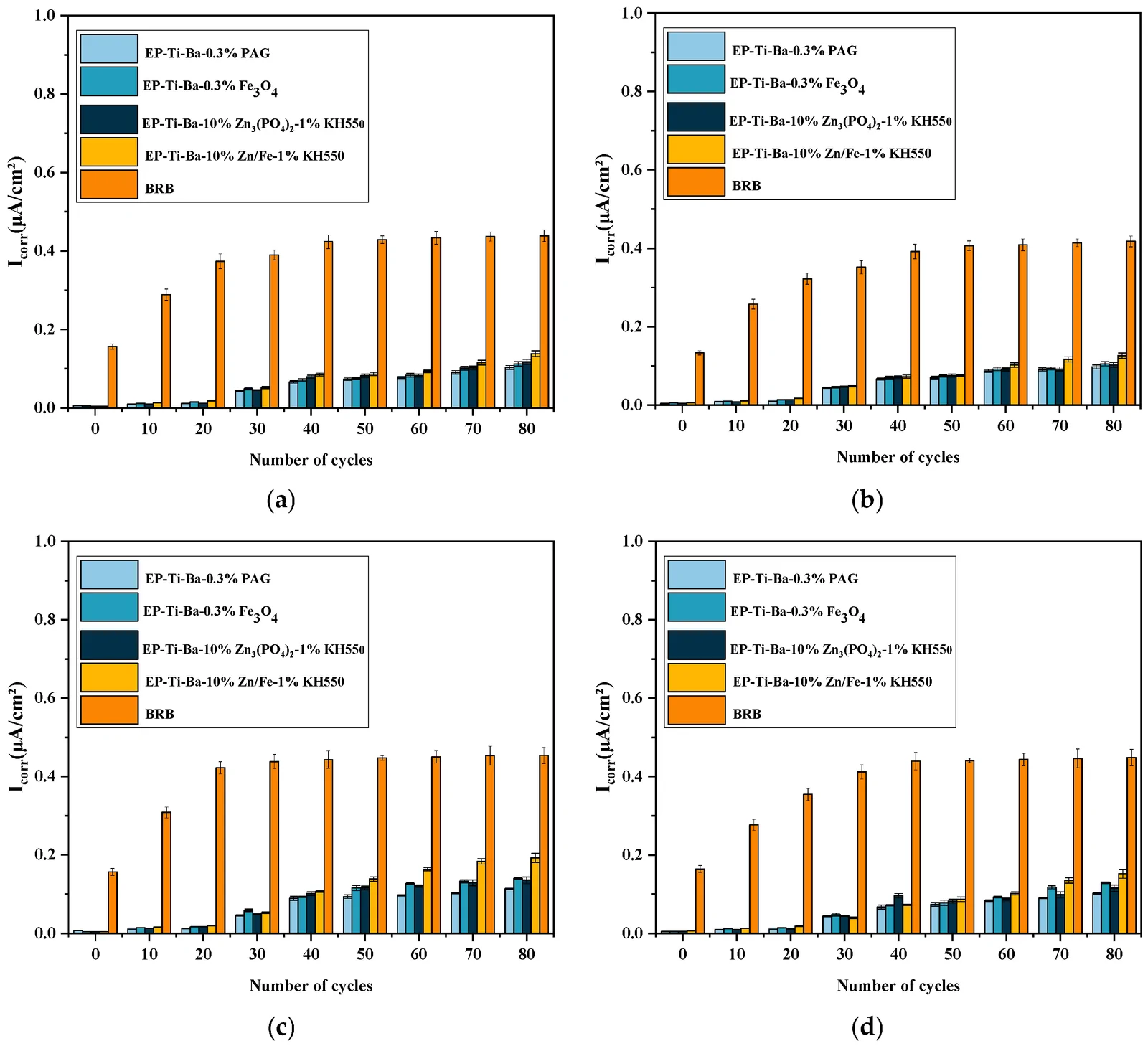

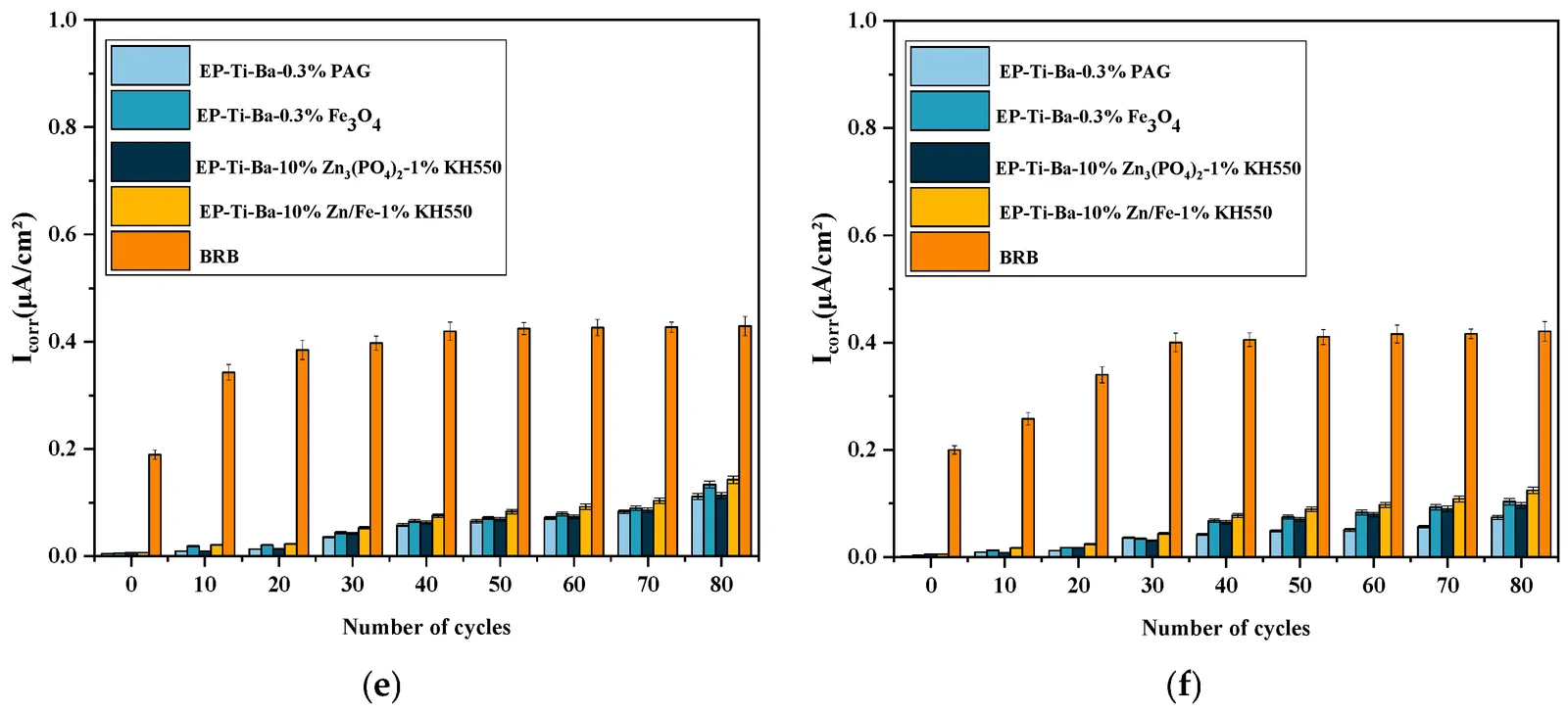
Corrosion current density results for coated reinforced concrete in saline soil environments: (**a**) C45-45c-VIE-50; (**b**) C45-50c-VIE-100; (**c**) C45-45c-VIE-2-50; (**d**) C45-50c-VIE-2-100; (**e**) C50-55c-VIF-1-50; (**f**) C50-60c-VIF-1-100.

Figure 4 shows the variation in corrosion current density (I_corr_) as a function of the number of cycles for bare reinforcing bars, graphene–polyaniline-coated reinforcing bars, nano-magnetite-coated reinforcing bars, zinc phosphate-coated reinforcing bars and zinc–iron powder-coated reinforcing bars under simulated wet–dry cycling conditions in saline soil. The data show that the corrosion current density of all coated reinforcing bars is significantly lower than that of the bare reinforcing bar. The corrosion current density of the unprotected bare reinforcing bar already exceeded 0.1 μA/cm^2^ at the initial stage (e.g., the initial value for C45-50c-VIE-1-50 was 0.1567 μA/cm^2^), indicating that the reinforcing bar was already in a state of activated corrosion. After 80 cycles, the corrosion current density rose further to 0.42–0.45 μA/cm^2^ (e.g., C45-45c-VIE-2-50 reached 0.4541 μA/cm^2^), indicating that the extent of corrosion had intensified.

Taking a test specimen with concrete strength C45 and a cover thickness of 50 mm as an example, in a chloride-dominated environment (VI-E-2, with a Cl^−^ concentration of 10,000 mg/L), the initial corrosion current density of bare reinforcing bars was 0.1641 μA/cm^2^; after 80 cycles, this rose to 0.4489 μA/cm^2^; whereas the I_corr_ of graphene–polyaniline-coated reinforcing bars increased only from 4.970 × 10^−3^ μA/cm^2^ to 0.1021 μA/cm^2^, remaining at a consistently low corrosion level. In a sulphate-dominated environment (VI-E-1, SO_4_^2−^ 10,000 mg/L), the corrosion current density of bare reinforcing bars after 80 cycles was slightly lower than that in the chloride-dominated environment (C45-50c-VIE-1-100, reaching 0.4179 μA/cm^2^). This phenomenon may be attributed to sulphate ions penetrating the concrete and reacting with Ca(OH)_2_ to form products that block the internal pores of the concrete, thereby reducing the extent of corrosion of the reinforcing steel [30,31]. The nano-Fe_3_O_4_ coating deteriorates most severely in a sulphate–chloride coupled environment, and the mechanism of its interface failure can be explained by the following three factors [32,33]: (1) When sulphate ions penetrate the concrete, they react with Ca(OH)_2_ to form expansive products such as ettringite, causing the concrete to crack and compromising the integrity of the coating–reinforcement interface. (2) In high-sulphate environments, nano-Fe_3_O_4_ may undergo oxidation or dissolution (Fe_3_O_4_ → Fe^3+^/Fe^2+^), thereby weakening its physical barrier function. (3) The accumulation of corrosion products (such as FeSO_4_) creates an acidic microenvironment at the interface, accelerating the process of synergistic corrosion. The combined effect of these multiple factors results in a significant decline in the protective performance of the Fe_3_O_4_ coating in sulphate-dominated environments, with a significant reduction in the coating’s physical and chemical protective capabilities [34,35]. It is worth noting that certain coatings (such as zinc phosphate) exhibit a phenomenon where I_corr_ first increases and then decreases in high-salt environments. It is hypothesised that this is due to the following mechanism: in the early stages of corrosion, harmful ions penetrate the substrate through microporosity in the coating, causing I_corr_ to rise; as corrosion products gradually block the pores, ion migration is impeded, and the corrosion rate consequently decreases. In summary, coatings can effectively delay the corrosion of reinforcing bars in saline-soil environments; however, there are significant differences in the resistance of different coatings to chloride and sulphate salts, with graphene–polyaniline exhibiting the best stability in mixed-salt environments.

### 4.5. Corrosion Current Density of Reinforcing Bars in Concrete Mixed with Seawater

To evaluate the effectiveness of epoxy coatings in enhancing the corrosion resistance of reinforcing bars in seawater–seasand concrete, corrosion resistance tests were conducted on coated reinforcing bars embedded in concrete mixed with seawater, and the performance differences of water-based epoxy coatings containing different fillers were compared. The mix proportions of the artificial seawater concrete are shown in Table 15.

**Figure 5:**
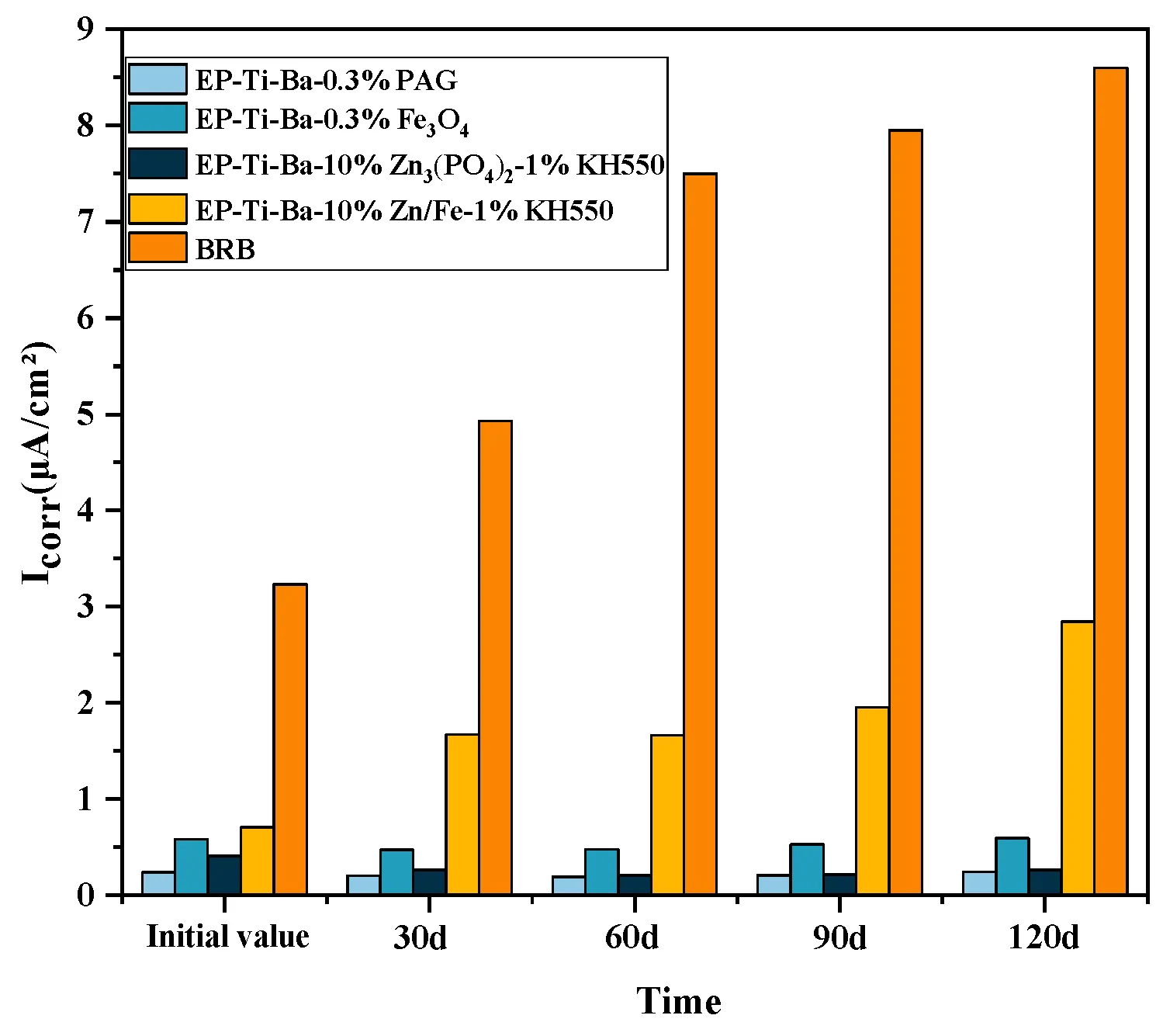
Time-dependent variation in the corrosion current density of test specimen C40-HS.

**Figure 6:**
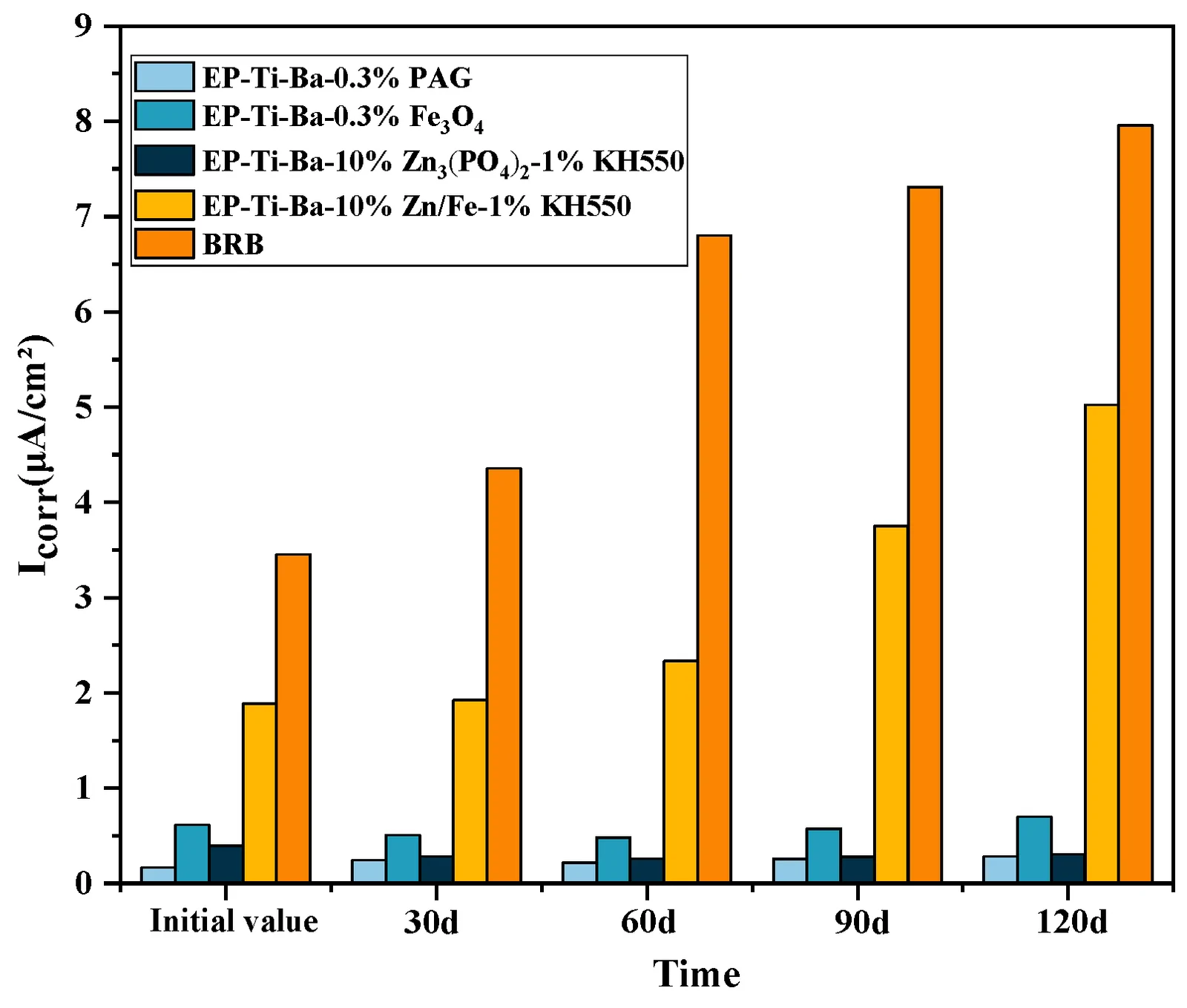
Time-dependent variation in the corrosion current density of test specimen C50-HS.

Figure 5 and Figure 6 show the time-dependent changes in I_corr_ for bare reinforcing bars and reinforcing bar electrodes coated with four types of homemade water-based epoxy coatings in seawater-mixed concrete. As can be seen from these two figures, the initial corrosion current density of the bare steel reinforcement concrete specimens, for which no anti-corrosion measures were taken, far exceeded 1 μA·cm^−2^ after demoulding, indicating that the reinforcement was already in a state of severe corrosion. However, the corrosion current density of the coated steel reinforcement electrodes was far lower than that of the bare steel reinforcement, demonstrating that the coating provided effective protection for the reinforcement. For example, the time-dependent variation in corrosion current density of specimen C40-HS shown in Figure 5 reveals that the initial corrosion current density of the bare reinforcing steel was 3.23 μA·cm^−2^. After 120 days, I_corr_ increased to 8.60 μA·cm^−2^, indicating that the reinforcing steel had reached a state of severe corrosion; in contrast, the I_corr_ values of the four coated reinforcing steel types after 120 days were far lower than that of the bare reinforcing steel. The corrosion current density was lowest for the coating containing 0.3% PAG nanocomposite, at 0.24 μA·cm^−2^, whilst the corrosion current density of the coated steel bar containing 10% Zn_3_(PO_4_)_2_ was the closest, at 0.26 μA·cm^−2^.

It should be noted that the initial I_corr_ values for exposed reinforcing bars in seawater-mixed concrete are relatively high (3.23 μA·cm^−2^ for C40-HS and 3.25 μA·cm^−2^ for C50-HS, as shown in Figure 5 and Figure 6); this is due to the direct penetration of chloride ions via the seawater used in the mix. Unlike in ordinary concrete, where chloride ions gradually permeate from the external environment, in seawater-mixed concrete, chloride ions are present in the pore solution from the very start of hydration. This higher internal chloride ion concentration disrupts the passivation film on the steel surface during curing, leading to measurable corrosion activity immediately after demoulding. This also explains why, even at the initial test point (Day 0, following a 28-day curing period), the I_corr_ value of the bare reinforcing bars had already exceeded 1 μA·cm^−2^. In contrast, the coated reinforcing bars maintained an I_corr_ value below 0.4 μA·cm^−2^ throughout the entire test, confirming the effectiveness of the water-based epoxy coating in protecting reinforcing bars in a chloride-containing environment.

### 4.6. Analysis of Case Studies on the Prediction of Reinforced Concrete Service Life Under Alternating Wet and Dry Seawater Conditions

**Figure 7:**
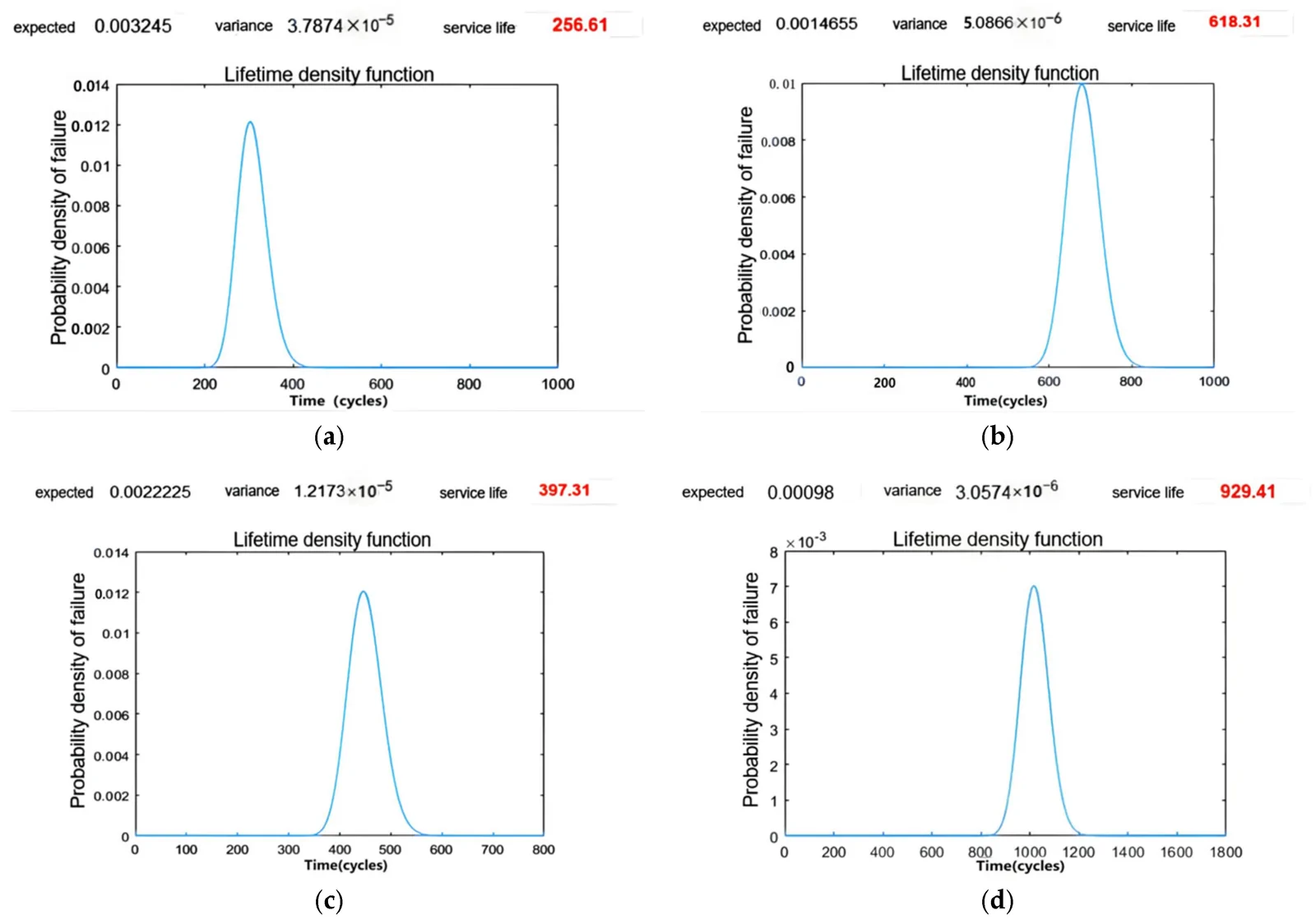
Results of the service life prediction for reinforced concrete under wet–dry cycles in a seawater environment. (**a**) Results of the service life prediction for uncoated concrete of the C50-60c-IIIF-50 mix in a cyclic wet–dry marine environment. (**b**) Results of the service life prediction for reinforced concrete coated with the C50-60c-IIIF-50 water-based epoxy coating in a cyclic wet–dry marine environment. (**c**) Results of the service life prediction for uncoated concrete of the C50-60c-IIIF-100 mix in a cyclic wet–dry marine environment. (**d**) Results of the service life prediction for the C50-65c-IIIF-100 water-based epoxy reinforced concrete mixture in a cyclic wet–dry marine environment.

Based on the test results for corrosion current density in coated and uncoated reinforced concrete under wet–dry cycling conditions, as described in Section 4.2, service life predictions were made for the C50-60c-IIIF-50 and C50-65c-IIIF-100 groups of coated and uncoated reinforced concrete. Figure 7 shows the service life prediction results for reinforced concrete: Under a seawater wet–dry cycling environment, for C50 concrete (with a 60 mm protective layer thickness), when the corrosion current density threshold reaches 1.0 μA·cm^−2^, the predicted service life of the uncoated reinforced concrete is 256 cycles, whilst that of the water-based epoxy-coated reinforced concrete is 618 cycles. According to the cycling regime described in Chapter 4, one cycle of the indoor accelerated seawater wet–dry cycling test is equivalent to one month of actual environmental exposure. Based on this, the service life of the bare reinforced concrete in this group is calculated to be 21.33 years, whilst that of the water-based epoxy-coated reinforced concrete is 51.50 years. By the same token, it can be predicted that when the C50 concrete cover thickness is 65 mm, the service life of bare reinforced concrete is 33 years, and that of water-based epoxy-coated reinforced concrete is 77.45 years. Consequently, for concrete with a design life of 50 years, under a seawater wet–dry cycling environment, and provided that the design requirements for concrete strength, minimum cover thickness and water-to-binder ratio specified in the standard are met, the use of water-based epoxy-coated reinforcing bars alone is projected to satisfy the design requirements. However, for concrete with a design life of 100 years, additional protective measures must be taken in addition to the use of water-based epoxy-coated reinforcing bars.

### 4.7. Analysis of Case Studies on the Prediction of Reinforced Concrete Service Life in De-Icing Salt Environments

**Figure 8:**
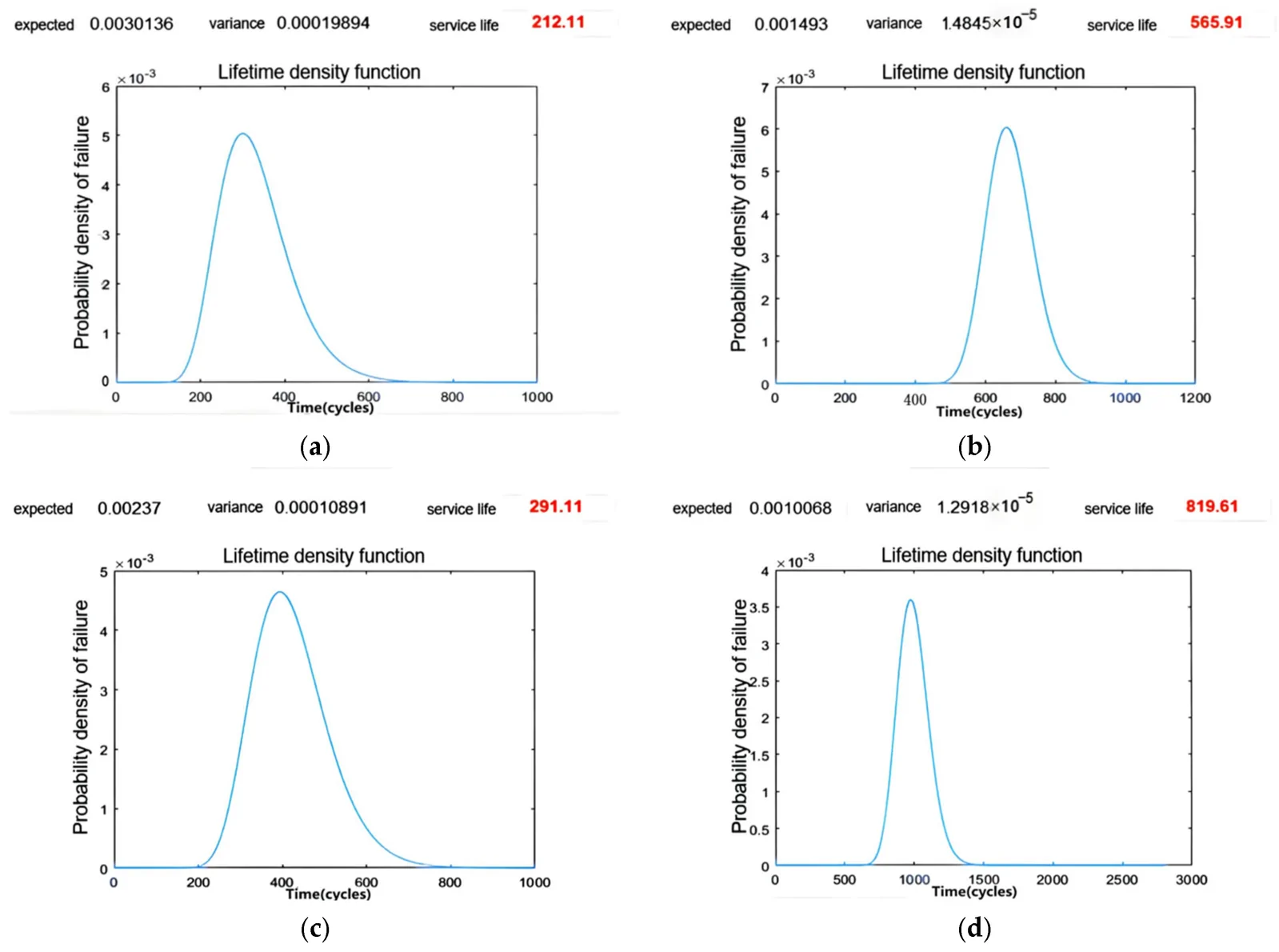
Results of the service life prediction for reinforced concrete in de-icing salt environments. (**a**) Results of the service life prediction for uncoated reinforced concrete of the C50-60c-IVF-50 class in de-icing salt environments. (**b**) Results of the service life prediction for reinforced concrete with water-based epoxy coatings of the C50-60c-IVF-50 group in de-icing salt environments. (**c**) Results of the service life prediction for uncoated reinforced concrete of the C50-65c-IVF-100 group in de-icing salt environments. (**d**) Results of the service life prediction for reinforced concrete with water-based epoxy coatings of the C50-65c-IVF-100 group in de-icing salt environments.

Based on the test results for corrosion current density in coated reinforced concrete under de-icing salt conditions presented in Section 4.3, service life predictions have been made for coated reinforced concrete and exposed reinforced concrete under the two service conditions C50-60c-IVF-50 and C50-65c-IVF-100. Figure 8 shows the service life prediction results for reinforced concrete under the C50-60c-IVF-50 conditions: In a de-icing salt environment, when the chloride ion concentration is 20,000 mg/L, for C50 concrete (with a 60 mm protective layer thickness), when the corrosion current density threshold reaches 1.0 μA·cm^−2^, the predicted service life of exposed reinforced concrete is 212.11 cycles, whilst that of water-based epoxy-coated reinforced concrete is 565.91 cycles. For C50 concrete (with a 65 mm protective layer thickness), when the corrosion current density threshold reaches 1.0 μA·cm^−2^, the predicted service life of uncoated reinforced concrete is 291.11 cycles, whilst that of water-based epoxy-coated reinforced concrete is 819.61 cycles. As one cycle in an indoor accelerated test is equivalent to 14 actual cycles and the annual number of freeze–thaw cycles for hydraulic concrete structures is 100, the service life of reinforced concrete can be inferred [36,37]. It can thus be deduced that under the C50-60c-IVF-50 conditions, the service life of bare reinforced concrete is 29 years, whilst that of water-based epoxy-coated reinforced concrete is 79 years; under the C50-65c-IVF-100 conditions, the service life of exposed reinforced concrete is 40 years, whilst that of water-based epoxy-coated reinforced concrete is 114 years. Consequently, in de-icing salt environments, provided that the design requirements for concrete strength, minimum cover thickness and water-to-binder ratio specified in the standards are met, the use of water-based epoxy-coated reinforced concrete alone is projected to satisfy the design requirements.

### 4.8. Analysis of Case Studies on the Prediction of Reinforced Concrete Service Life in Saline-Alkali Soils

**Figure 9:**
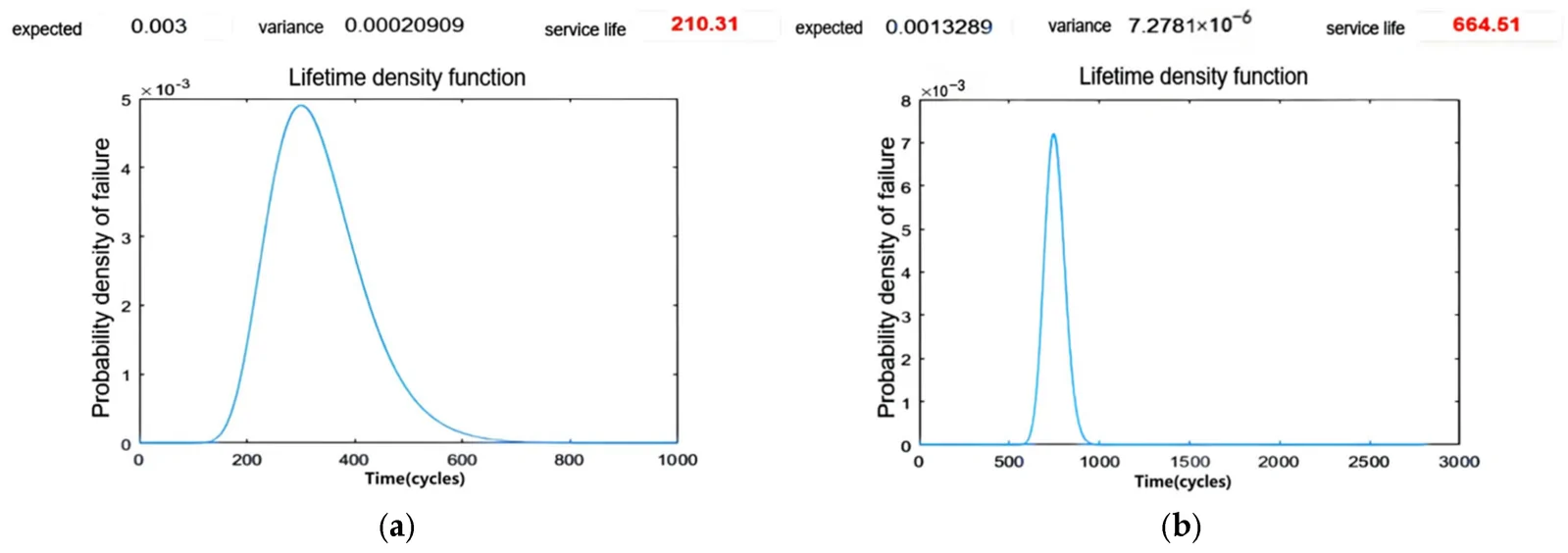

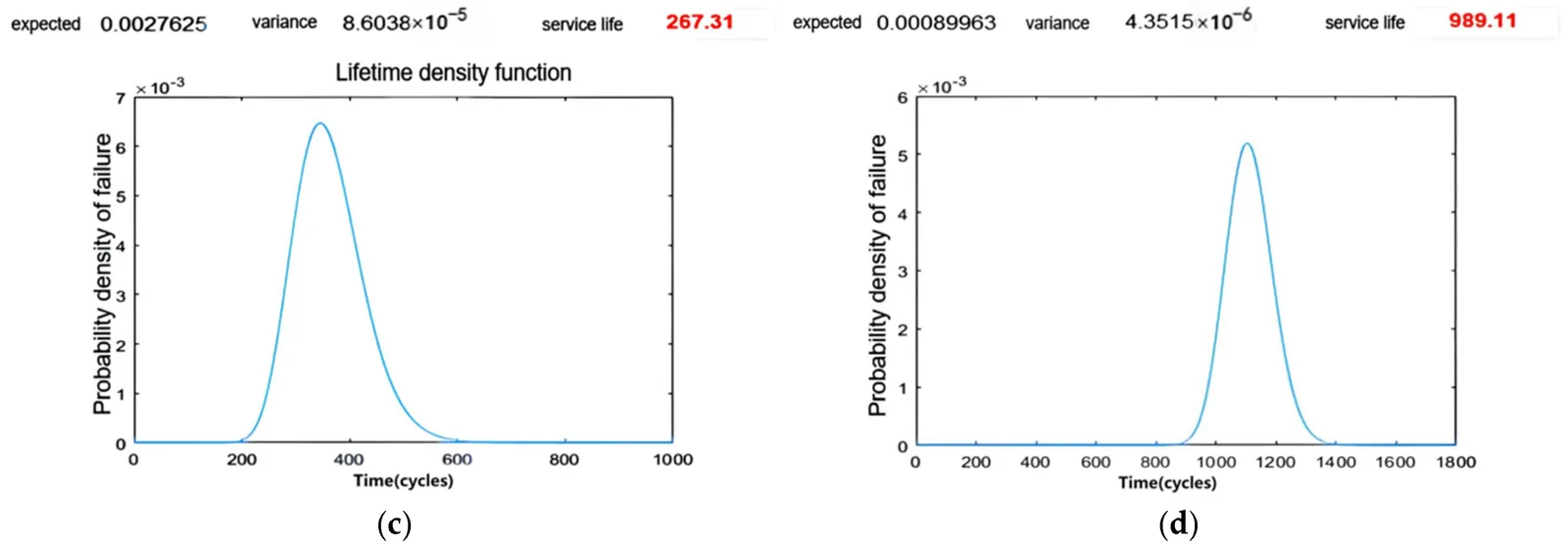
Results of the service life prediction for reinforced concrete in saline-alkali soil environments. (**a**) Results of the service life prediction for exposed reinforced concrete under the C50-55c-VIF-1-50 conditions in saline-alkali soil environments. (**b**) Results of the service life prediction for water-based epoxy-coated reinforced concrete under the C50-55c-VIF-1-50 conditions in saline soil environments. (**c**) Results of service life predictions for uncoated, exposed reinforced concrete of the C50-60c-VIF-1-100 group in saline soil environments. (**d**) Results of the service life prediction for reinforced concrete coated with water-based epoxy coatings under the C50-60c-VIF-1-100 conditions in saline soil environments.

Based on the results of the corrosion current density tests on coated reinforced concrete under saline soil conditions as described in Section 4.4, service life predictions were made for coated reinforced concrete and exposed reinforced concrete under the test conditions C50-55c-VIF-1-50 and C50-60c-VIF-1-100. Figure 9 shows the service life predictions for reinforced concrete under the C50-55c-VIF-1-50 and C50-60c-VIF-1-100 conditions: in a saline soil environment, when the chloride ion concentration is 2000 mg/L, and the sulphate ion concentration is 15,000 mg/L, for C50 concrete (with a 55 mm protective layer thickness), when the corrosion current density threshold reaches 1.0 μA·cm^−2^, the predicted service life of bare reinforced concrete is 210.31 cycles, whilst that of water-based epoxy-coated reinforced concrete is 664.51 cycles. For C50 concrete (60 mm protective layer thickness), when the corrosion current density threshold reaches 1.0 μA·cm^−2^, the predicted service life of uncoated reinforced concrete is 267.31 cycles, whilst that of reinforced concrete with a water-based epoxy coating is 989.11 cycles. The acceleration factor for indoor accelerated tests in a saline soil environment is 10; from this, it can be inferred that for the C50-55c-VIF-1-50 group, the service life of uncoated reinforced concrete is 22 years, whilst that of water-based epoxy-coated reinforced concrete is 81 years [38]. By the same token, it can be predicted that the service life of the C50-60c-VIF-1-100 group of bare reinforced concrete is 22 years, whilst that of water-based epoxy-coated reinforced concrete is 81 years. Consequently, for concrete with a design life of 50 years in a saline soil environment, provided that the design requirements for concrete strength, minimum cover thickness and water-to-binder ratio specified in the standards are met, the use of water-based epoxy-coated reinforcing bars alone is projected to satisfy the design requirements. However, for concrete with a design life of 100 years, additional protective measures must be taken in addition to the use of water-based epoxy-coated reinforcing bars.

## 5. Conclusions

This paper systematically investigates the corrosion resistance of reinforced concrete coated with four types of water-based epoxy coatings under various severe conditions, including long-term immersion in marine chlorides combined with wet–dry cycling, de-icing salt freeze–thaw cycling, salt-saturated soil drying and soaking cycles, and concrete mixed with seawater. Based on the time-dependent evolution of corrosion current density (I_corr_), the study elucidated the protective mechanisms and failure modes of the different coatings, and employed the Wiener random process model to predict their service life. The main conclusions are as follows:

(1) Water-based epoxy coatings can significantly enhance the corrosion resistance of reinforcing bars in harsh environments, with the corrosion current density of all four coated reinforcing bars generally reduced by more than 70 per cent. Through a multi-mechanism synergistic effect—comprising physical shielding, chemical passivation by functional fillers, and dynamic blocking of micropores by corrosion products—the coating effectively maintains the passivated state of the reinforcing bar surface. Among these, the nanocomposite coating containing 0.3% graphene–polyaniline (PAG) exhibited the best overall protective performance. Owing to the synergistic enhancement provided by the two-dimensional physical barrier of graphene and the doping/dedoping effects of polyaniline, it demonstrated the lowest corrosion current density and the slowest degradation rate across all environments.
(2) Different coatings exhibit significant selective sensitivity to the types of corrosive ions. Zinc phosphate coatings form a ‘dynamic pore-blocking effect (hypothesised)’ barrier through the dynamic pore-blocking effect of corrosion products such as zinc hydroxychloride and iron phosphate, demonstrating outstanding stability in de-icing salt environments with high chloride concentrations. The nano-sized iron(III) oxide (Fe_3_O_4_) coating deteriorated most severely in a sulphate–chloride coupled environment, which can be attributed to the delamination at the coating–substrate interface caused by the expansive stresses of sulphate corrosion products, as well as the oxidation and dissolution of Fe_3_O_4_; the sacrificial anode protection provided by zinc–iron powder was limited by the rapid consumption of zinc.
(3) Service life predictions based on the Wiener process suggest that, provided the specifications for concrete strength class, cover thickness and water-to-binder ratio are met, water-based epoxy coatings are projected to delay the onset of severe corrosion (I_corr_ ≥ 1 μA·cm^−2^) beyond the 50-year design threshold in seawater wet–dry cycling zones and saline soil environments, and are expected to meet the 100-year design requirements in de-icing salt environments. However, as these predictions are derived from accelerated laboratory tests, they should be interpreted as indicative estimates rather than definitive guarantees. Long-term field validation is necessary to confirm the actual service life under real service conditions. However, for structures in seawater wet–dry cycling zones and saline soil environments with a 100-year design service life, it is recommended to adopt a multi-component synergistic protection strategy comprising ‘high-performance concrete plus epoxy coating’.

It should be acknowledged that this study has one limitation. Although the differences in corrosion current density observed across the five test formulations provide clear evidence of their relative protective performance, the mechanistic explanations underlying these findings—including barrier effects, electrochemical passivation, pore blockage by corrosion products, and sacrificial anode behaviour—are based primarily on precedents in the literature rather than on direct experimental characterisation of the coatings following exposure. To verify the presence and role of these mechanisms in the specific formulations tested, techniques such as scanning electron microscopy–energy dispersive spectroscopy (SEM–EDS), X-ray diffraction (XRD), Raman spectroscopy, X-ray photoelectron spectroscopy (XPS) and cross-sectional analysis are required. We have explicitly identified this as a key direction for future research.

## Figures and Tables

**Table 1:** Main chemical composition of HRB400 reinforcing bars.

Ingredients	C	Si	Mn	P	S	Ceq
Content (%)	0.248	0.289	0.948	0.018	0.031	0.431

**Table 2:** Design of coating mix proportions.

Group	Mix Proportions
1	Water-based epoxy emulsion + titanium dioxide + precipitated barium sulphate + 0.3% graphene–polyaniline EP-Ti-Ba-0.3%PAG
2	Water-based epoxy emulsion + titanium dioxide + precipitated barium sulphate + 0.3% iron oxide powder EP-Ti-Ba-0.3%Fe_3_O_4_
3	Water-based epoxy emulsion + titanium dioxide + precipitated barium sulphate + 10% zinc phosphate + 1% silane solution EP-Ti-Ba-10%Zn_3_(PO_4_)_2_-1%KH550
4	Water-based epoxy emulsion + titanium dioxide + precipitated barium sulphate + 10% zinc–iron powder + 1% silane solution EP-Ti-Ba-10% Zn/Fe-1%KH550
5	Bare reinforcing bars BRBs

**Table 3:** Main components and concentrations of artificial seawater.

Constituent	Concentration/(g·L^−1^)	Constituent	Concentration/(g·L^−1^)
NaCl	24.530	NaHCO_3_	0.201
MgCl_2_	5.200	KBr	0.101
Na_2_SO_4_	4.090	H_3_BO_3_	0.027
CaCl_2_	1.160	SrCl_2_	0.025
KCl	0.695	NaF	0.003

**Table 4:** Concrete mix proportions (kg/m^3^) and 28-day compressive strength.

Concrete Strength Class	Water-to- Binder Ratio	Water	Cement	Mineral Powder	Fly Ash	Sand	Aggregate	Water-Reducing Agent	28-Day Compressive Strength (MPa)
C40	0.42	185	220	154	66	620	1150	1.35	45.6
C45-0.40	0.40	180	225	157.5	67.5	620	1150	1.56	53.2
C45-0.38	0.38	175	230	161	69	620	1150	2.07	55.8
C45-0.36	0.36	170	236	166	70	620	1150	2.36	57.4
C50	0.36	160	222.5	155.5	67	630	1220	2.67	58.9
C50	0.34	151	222.5	155.5	67	630	1220	2.67	62.3
C60	0.32	128	200	140	60	709	1064	3.2	68.5
C70	0.28	127	227	159	68	615	1094	5.45	76.8

**Table 5:** Levels of impact of marine chloride environments [16].

Exposure Class	Environmental Conditions	Examples of Concrete Structural Members
III-C	Moderate	Bridge piers, pile caps, foundations
III-D	Severe	Bridge piers, superstructure components; external walls and external structural elements of coastal land-based buildings
III-E	Very	Bridge piers, superstructure components; external walls and external structural elements of coastal land-based buildings
III-F	Extreme	Bridge piers, pile caps, quays

**Table 6:** Minimum cover thickness, c (mm), for concrete and reinforcing steel in chloride environments [11].

	Design Service Life	100 Years			50 Years			30 Years		
Environmental Impact Level		Concrete Strength Class	Maximum Water-to-Cement Ratio	c	Concrete Strength Class	Maximum Water-to-Cement Ratio	c	Concrete Strength Class	Maximum Water-to-Cement Ratio	c
Flat components such as panels and walls	III-C	C45	0.4	45	C40	0.42	40	C40	0.42	35
	IV-C	C45	0.4	55	C40	0.42	50	C40	0.42	45
	III-D	≥C50	0.36	50	≥C45	0.40	45	≥C45	0.40	40
	III-D	C50	0.36	60	C45	0.40	55	C45	0.40	45
	III-E	≥C55	0.33	55	≥C50	0.36	50	≥C50	0.36	40
	IV-E	C45	0.36	65	C50	0.36	60	C50	0.36	55
	III-F	C45	0.33	60	≥C55	0.36	55	C50	0.36	55
Rectangular members such as beams and columns	III-C	C45	0.4	50	C40	0.42	45	C40	0.42	40
	IV-C	C45	0.4	60	C40	0.42	55	C40	0.42	50
	III-D	≥C50	0.36	55	≥C45	0.40	50	≥C45	0.40	40
	IV-D	C50	0.36	65	C45	0.40	60	C45	0.40	50
	III-E	≥C55	0.33	60	≥C50	0.36	55	≥C50	0.36	45
	IV-E	C45	0.36	70	C50	0.36	65	C50	0.36	55
	III-F	C45	0.33	65	≥C55	0.36	60	C50	0.36	55

**Table 7:** Design of a test programme for the durability of reinforced concrete with water-based epoxy coatings in marine chloride environments.

Specimen Number	Concrete Strength Class	Corrosive Medium	Maximum Water-to-Binder Ratio	Protective Layer	Number of Test Specimens (Pieces)
C40-40c-IIIC-50	C40	Artificial seawater	0.42	40	5
C45-45c-IIIC-100	C45		0.4	45	5
C50-65c-IIIF-100	C50		0.36	65	5
C50-60c-IIIF-50	C50		0.36	60	5

**Table 8:** Dry–wet cycling regimen.

Month	Temperature/°C	Dry-to-Wet Ratio	Ratio of Dry to Wet Time per Cycle/h
1, 2, 3, 12	20	9:1	65:7
4, 5, 10, 11	30	14:1	67:5
6, 7, 8, 9	40	6:1	62:10

**Table 9:** Impact levels in de-icing salt environments [16,18].

Environmental Impact Level	Environmental Impact Intensity	Reference Environmental Conditions	Reference Chloride Ion Concentration
IV-C	Moderate	1. Subject to mild exposure to salt spray from de-icing salt; 2. Submerged on all sides in water containing chlorides; 3. Exposure to water containing low concentrations of chloride ions, with alternating wet and dry conditions	Low (100 mg/L~500 mg/L)
IV-D	Severe	1. Subject to light splashing from de-icing brine; 2. Exposure to water containing high concentrations of chloride ions, with alternating wet and dry conditions	Moderately high (500 mg/L~5000 mg/L)
IV-E	Very severe	1. Direct contact with de-icing salt solutions; 2. Exposure to heavy splashes or heavy salt spray from de-icing salt solutions; 3. Contact with water containing high concentrations of chloride ions, with alternating wet and dry conditions	High (greater than 5000 mg/L)
IV-F	Extremely severe	1. Direct contact with de-icing salt solutions; 2. Exposure to heavy splashes or heavy salt spray from de-icing salt solutions; 3. Contact with water containing extremely high concentrations of chloride ions, with alternating wet and dry conditions	Extremely high (greater than 10,000 mg/L)

**Table 10:** Test protocol for the durability of epoxy-coated reinforced concrete in de-icing salt environments.

Specimen Number	Concrete Strength Class	Chloride Ion Concentration (mg/L)	Maximum Water-to-Cement Ratio	Cover Thickness (mm)	Number of Test Specimens (Pieces)
C45-55c-IVD-100	C45	5000	0.40	55	5
C45-45c-IVD-50	C45	5000	0.40	45	5
C50-60c-IVE-100	C50	10,000	0.36	60	5
C50-50c-IVE-50	C50	10,000	0.36	50	5
C50-65c-IVF-100	C50	20,000	0.36	65	5
C50-60c-IVF-50	C50	20,000	0.36	60	5

**Table 11:** Environmental impact levels of saline-alkali soils [17,18].

Environmental Impact Level	Classification of Chloride Ion Concentrations for Proposed Trials	Classification of Sulphate Ion Concentrations for the Proposed Experiment	Guidelines for Classifying Chloride Ion Concentrations	Guidelines for Classifying Sulphate Ion Concentrations
VI-C	2000 mg/L	2000 mg/L	1000 mg/L~2000 mg/L	1000 mg/L~2000 mg/L
VI-D	2000 mg/L	5000 mg/L	1000 mg/L~2000 mg/L	2000 mg/L~5000 mg/L
VI-E-1	2000 mg/L	10,000 mg/L	1000 mg/L~2000 mg/L	5000 mg/L~10,000 mg/L
VI-E-2	10,000 mg/L	2000 mg/L	5000 mg/L~10,000 mg/L	1000 mg/L~2000 mg/L
VI-F-1	2000 mg/L	15,000 mg/L	1000 mg/L~2000 mg/L	10,000 mg/L~15,000 mg/L
VI-F-2	15,000 mg/L	2000 mg/L	10,000 mg/L~15,000 mg/L	1000 mg/L~2000 mg/L
VI-H	35,000 mg/L	20,000 mg/L	/	/

**Table 12:** Minimum concrete strength class, maximum water-to-binder ratio, and minimum cover thickness in saline-alkali soil environments.

Years of Design Experience (Years)	Sulphate Concentration Range (mg/L)	Range of Chloride Concentration (mg/L)	Minimum Strength Class	Maximum Water-to-Binder Ratio	Minimum Protective Layer Thickness (mm)
50	10,000~14,000	1000~5000	C45	0.38	50
		5001~20,000	C45	0.36	55
		20,001~50,000	C50	0.36	55
	14,001~17,000	1000~5000	C50	0.36	55
		5001~20,000	C50	0.34	55
		20,001~50,000	C55	0.34	60
	17,001~20,000	1000~5000	C55	0.34	60
		5001~20,000	C55	0.32	60
		20,001~50,000	C60	0.32	65
100	10,000~14,000	1000~5000	C45	0.36	55
		5001~20,000	C50	0.36	55
		20,001~50,000	C50	0.36	60
	14,001~17,000	1000~5000	C50	0.34	60
		5001~20,000	C55	0.34	60
		20,001~50,000	C55	0.34	65
	17,001~20,000	1000~5000	C55	0.32	65
		5001~20,000	C60	0.32	65
		20,001~50,000	C70	0.28	70

**Table 13:** Test protocol for the durability testing of epoxy-coated reinforced concrete in saline soil environments.

Specimen Number	Concrete Strength Class	Water-to-Cement Ratio	Thickness of Protective Layer (mm)	Number of Test Specimens (Pieces)
C45-50c-VIE-1-100	C45	0.36	50	5
C45-45c-VIE-1-50	C45	0.36	45	5
C45-50c-VIE-2-100	C45	0.36	50	5
C45-45c-VIE-2-50	C45	0.36	45	5
C50-60c-VIF-1-100	C50	0.34	60	5
C50-55c-VIF-1-50	C50	0.34	55	5

**Table 14:** Relationship with the state of reinforcement corrosion.

$I_{\mathrm{corr}}$	Condition of Reinforcement Corrosion
$I_{\mathrm{corr}}\;<\;0.1\;\mu A\cdot {\mathrm{cm}}^{-2}$	Passivated state
$0.1\;\mu A\cdot {\mathrm{cm}}^{-2}\;<\;I_{\mathrm{corr}}\;<\;0.5\;\mu A\cdot {\mathrm{cm}}^{-2}$	Low corrosion state
$0.5\;\mu A\cdot {\mathrm{cm}}^{-2}\;<\;I_{\mathrm{corr}}\;<\;1\;\mu A\cdot {\mathrm{cm}}^{-2}$	Moderate corrosion state
$I_{\mathrm{corr}}\;>\;1\;\mu A\cdot {\mathrm{cm}}^{-2}$	Severe corrosion state

Footnote: I_corr_ > 1 μA·cm^−2^ indicates severe corrosion, which corresponds to a high level of corrosion activity requiring timely maintenance or protective measures.

**Table 15:** Mix proportions for concrete mixed with artificial seawater (kg/m^3^) and 28-day compressive strength.

Concrete Strength Class	Water-to-Binder Ratio	Artificial Seawater	Cement	Mineral Powder	Fly Ash	Sand	Gravel	Water-Reducing Agent	28-Day Compressive Strength (MPa)
C40	0.42	185	220	154	66	620	1150	1.35	47.9
C50	0.36	160	222.5	155.5	67	630	1220	2.67	58.5

## Data Availability

The original contributions presented in this study are included in the article. Further inquiries can be directed to the corresponding authors.
